# Protective prototype-Beta and Delta-Omicron chimeric RBD-dimer vaccines against SARS-CoV-2

**DOI:** 10.1016/j.cell.2022.04.029

**Published:** 2022-06-23

**Authors:** Kun Xu, Ping Gao, Sheng Liu, Shuaiyao Lu, Wenwen Lei, Tianyi Zheng, Xueyuan Liu, Yufeng Xie, Zhennan Zhao, Shuxin Guo, Cong Tang, Yun Yang, Wenhai Yu, Junbin Wang, Yanan Zhou, Qing Huang, Chuanyu Liu, Yaling An, Rong Zhang, Yuxuan Han, Minrun Duan, Shaofeng Wang, Chenxi Yang, Changwei Wu, Xiaoya Liu, Guangbiao She, Yan Liu, Xin Zhao, Ke Xu, Jianxun Qi, Guizhen Wu, Xiaozhong Peng, Lianpan Dai, Peiyi Wang, George F. Gao

**Affiliations:** 1 Research Network of Immunity and Health (RNIH), Beijing Institutes of Life Science, Chinese Academy of Sciences, Beijing 100101, China; 2 CAS Key Laboratory of Pathogen Microbiology and Immunology, Institute of Microbiology, Chinese Academy of Sciences, Beijing 100101, China; 3 University of Chinese Academy of Sciences, Beijing 100049, China; 4 Cryo-EM Center, Southern University of Science and Technology, Shenzhen 518055, China; 5 National Kunming High-level Biosafety Primate Research Center, Institute of Medical Biology, Chinese Academy of Medical Sciences and Peking Union Medical College, Kunming 650031, China; 6 NHC Key Laboratory of Biosafety, National Institute for Viral Disease Control and Prevention, Chinese Center for Disease Control and Prevention, Beijing 102206, China; 7 Zhejiang University School of Medicine, Hangzhou 310058, China; 8 School of Public Health, Cheeloo College of Medicine, Shandong University, Jinan 250012, China; 9 Department of Basic Medical Sciences, School of Medicine, Tsinghua University, Beijing 100084, China; 10 Faculty of Health Sciences, University of Macau, Macau, SAR 999078, China; 11 State Key Laboratory for Conservation and Utilization of Subtropical Agro-Bioresources, Guangxi University, Nanning 530004, China; 12 Savaid Medical School, University of Chinese Academy of Sciences, Beijing 101408, China; 13 School of Life Sciences, Yunnan University, Kunming 650091, China; 14 Anhui Zhifei Longcom Biopharmaceutical Co. Ltd, Hefei 230088, China; 15 Chongqing Medleader Bio-Pharm, Chongqing 401338, China; 16 State Key Laboratory of Medical Molecular Biology, Department of Molecular Biology and Biochemistry, Institute of Basic Medical Sciences, Medical Primate Research Center, Neuroscience Center, Chinese Academy of Medical Sciences, School of Basic Medicine, Peking Union Medical College, Beijing 100005, China

**Keywords:** SARS-CoV-2, COVID19 vaccine, variant of concern, VOC, Omicron variant, Delta variant, receptor-binding domain, RBD, vaccine protection, immunogen structure

## Abstract

Breakthrough infections by SARS-CoV-2 variants become the global challenge for pandemic control. Previously, we developed the protein subunit vaccine ZF2001 based on the dimeric receptor-binding domain (RBD) of prototype SARS-CoV-2. Here, we developed a chimeric RBD-dimer vaccine approach to adapt SARS-CoV-2 variants. A prototype-Beta chimeric RBD-dimer was first designed to adapt the resistant Beta variant. Compared with its homotypic forms, the chimeric vaccine elicited broader sera neutralization of variants and conferred better protection in mice. The protection of the chimeric vaccine was further verified in macaques. This approach was generalized to develop Delta-Omicron chimeric RBD-dimer to adapt the currently prevalent variants. Again, the chimeric vaccine elicited broader sera neutralization of SARS-CoV-2 variants and conferred better protection against challenge by either Delta or Omicron SARS-CoV-2 in mice. The chimeric approach is applicable for rapid updating of immunogens, and our data supported the use of variant-adapted multivalent vaccine against circulating and emerging variants.

## Highlights

• A chimeric RBD-dimer immunogenic approach can be used to rapidly adapt SARS-CoV-2 variants
• Chimeric RBD-dimers elicit broader responses to variants compared with homodimers
• Prototype-Beta chimeric vaccine is protective in both mice and macaques
• Delta-Omicron chimeric vaccine protects mice from either Delta or Omicron challenge

## Graphical abstract

**Figure:**
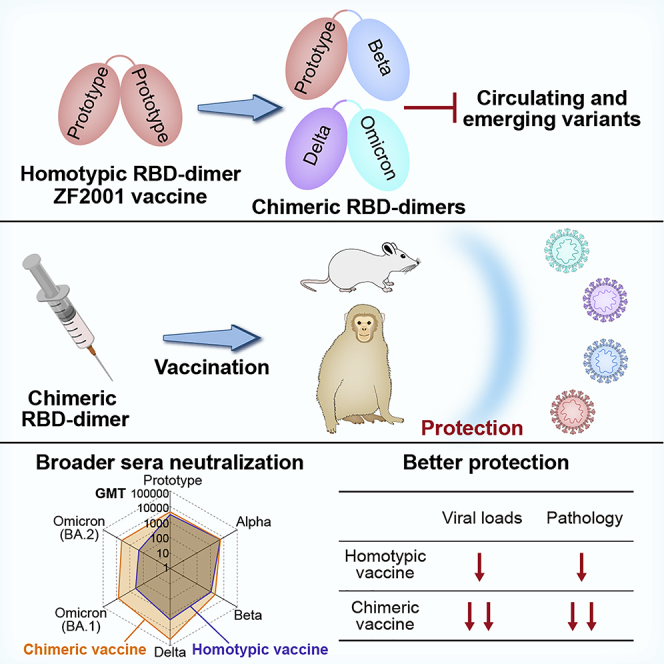

## Teaser

Vaccine immunogens designed as chimeras of the spike protein receptor-binding domain of two distinct SARS-CoV-2 variants elicit broad serum neutralization and protection from infection by the Beta, Delta, and Omicron variants in mice and macaques. The chimeric approach is applicable for rapid updating of immunogens against both circulating and emerging variants.

## Introduction

Severe acute respiratory syndrome coronavirus 2 (SARS-CoV-2) variants are continually emerging, and they become the circulating strains in the world (www.who.int). Several highly transmissible variants of concern (VOCs) showed altered pathogenicity and transmission (Karim and Karim, 2021; Krause et al., 2021; Viana et al., 2022) bringing new waves of infection worldwide. The immune escape of the variants to the current approved vaccines and, concomitantly, the breakthrough infections have become the global challenge to end the pandemic (Liu et al., 2021a). Currently, all approved vaccines are based on prototype SARS-CoV-2. Given the circulating strains of SARS-CoV-2 are fast changing, it is debated yet in the field that what our vaccine strategy is going forward? Should it be the prototype boosting, or using variant-specific vaccine, or using multivalent vaccine with broader coverage of both circulating and emerging variants?

Coronavirus spike protein mediated virus entry via its receptor-binding domain (RBD) (Dai and Gao, 2021; Wang et al., 2020). RBD is the major target for neutralizing antibodies (NAbs) and a favorable antigen for vaccine development because of its immunodominance (Dai and Gao, 2021). We previously designed novel vaccines against Beta coronavirus using tandem repeat dimeric RBD as antigens, which substantially enhanced immunogenicity in animal model (Dai et al., 2020). Based on this strategy, we developed protein subunit vaccine, ZF2001, against coronavirus diseases 2019 (COVID-19) (Yang et al., 2021), which has received conditional marketing authorization in China and emergency use authorization in Uzbekistan, Indonesia, and Columbia. ZF2001 is rolling out in the vaccination campaign with more than 250 million doses administrated globally. Phase 3 clinical trials showed that ZF2001 has more than 80% efficacy in preventing symptomatic COVID-19 (Dai et al., 2022). ZF2001 vaccine uses RBD sequence from Wuhan-Hu-1 strain (prototype) (Wei et al., 2020) and elicits antibody responses in human with varying neutralizing activities to SARS-CoV-2 variants (Huang et al., 2021; Zhao et al., 2021). Before the emergence of Omicron variant, Beta variant contains the most mutations in the spike protein and showed the largest reduction in sensitivity to vaccines or neutralizing monoclonal antibodies (mAbs) (Abdool Karim and de Oliveira, 2021; Garcia-Beltran et al., 2021; Liu et al., 2021b; Lucas et al., 2021; Shen et al., 2021; Tegally et al., 2021; Wall et al., 2021; Wang et al., 2021a, 2021b; Wibmer et al., 2021; Wu et al., 2021). Similar conclusion was also observed in vaccinees who received ZF2001 vaccine (Zhao et al., 2021). The recent occurring Omicron variant showed more severe immune escape than the Beta variant (Cameroni et al., 2022; Cele et al., 2022; Dejnirattisai et al., 2022; Liu et al., 2022; VanBlargan et al., 2022), and accordingly, the neutralizing activities of ZF2001-elicited human sera were 3.1- to 10.6-fold lower against recently circulating Omicron variant than prototype (Zhao et al., 2022). Given the positive correlation between neutralizing titer and protection efficacy (Khoury et al., 2021), the sera neutralization against SARS-CoV-2 variants is believed to impact vaccine effectiveness.

When the Beta emerged as the most resistant VOC in early 2021, we first generated prototype-Beta chimeric RBD-dimers to cover both prototype and Beta linages. The antigenic characterization and structural analysis of RBD-dimer immunogens were performed to validate the major epitopes displayed. The neutralizing activities of vaccine-elicited mice sera against a panel of SARS-CoV-2 variants were further tested. The protection efficacy was evaluated in mice. Given the outperformance of the prototype-Beta chimeric RBD-dimer in comparison with its homotypic counterparts, we produced the prototype-Beta chimeric antigen in pilot scale for preclinical efficacy study in rhesus macaques. Vaccine-elicited humoral and cellular immune responses were tested, and subsequently, the protective efficacies against challenge from prototype, Beta and Delta SARS-CoV-2, were further evaluated. Over the next few months, Delta and Omicron became the major circulating variants one after another. Vaccines inducing broader immune responses, with particularly high activity against both Delta and Omicron variants, are urgently needed. Therefore, using the chimeric approach, we rapidly developed the Delta-Omicron RBD-dimer vaccine. The antigenic integrity and cryoelectron microscopy (cryo-EM) structure were determined; the immunogenicity and protective efficacy were further evaluated, demonstrating the advantage and generalizability of the chimeric vaccine approach in countermeasure of SARS-CoV-2 variants. This is a proof of concept for rapid updating of immunogens based on the chimeric approach, and our data supported the WHO’s advice to develop multivalent and variant-adapted COVID-19 vaccines ensuring the breadth of the immune response against circulating and emerging variants (WHO, 2022).

## Results

### Development of prototype-Beta vaccine based on the chimeric RBD-dimer vaccine design strategy

**Figure S1:**
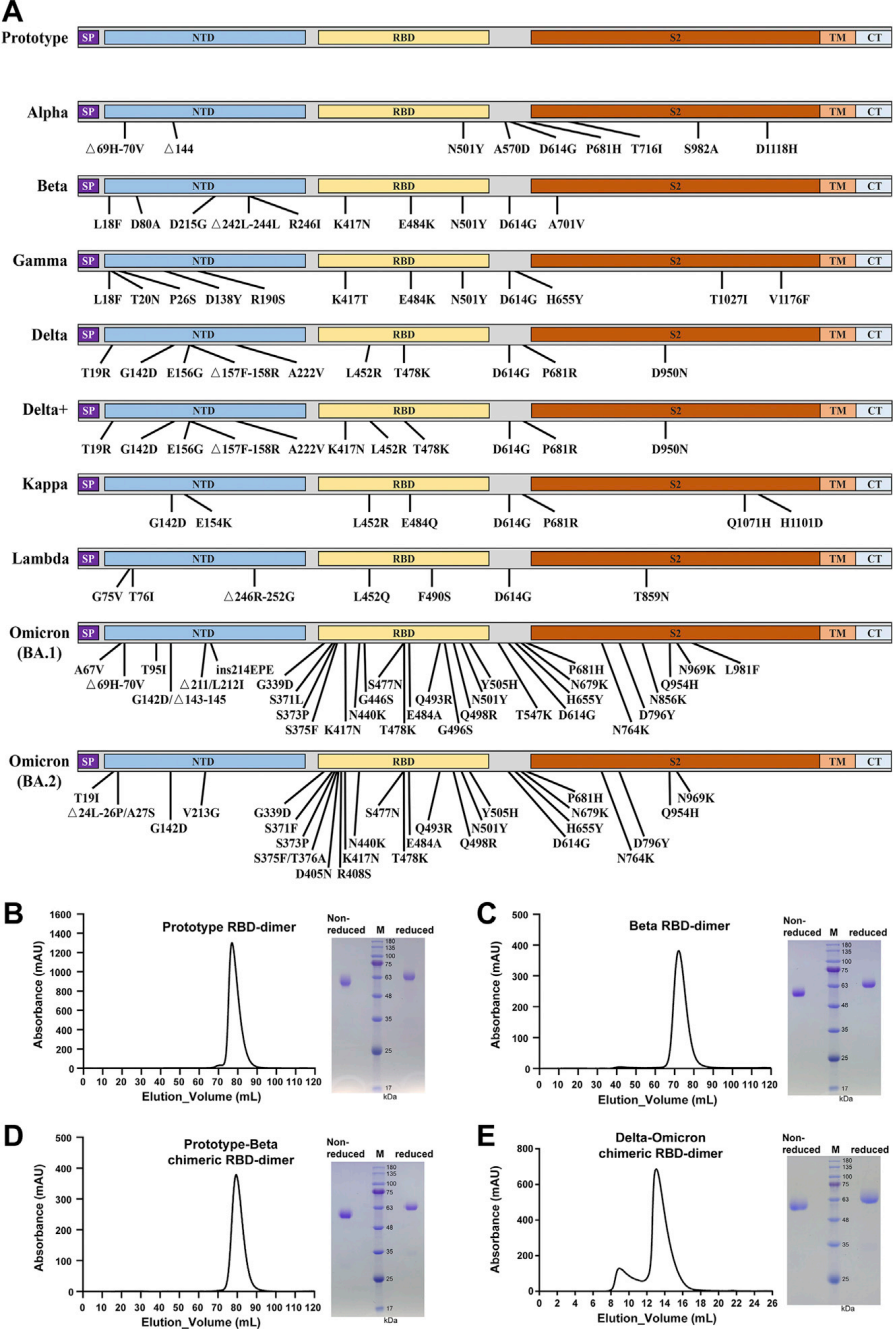
Schematic representation of SARS-CoV-2 spike proteins used in this study and analytical gel filtration profiles of RBD-dimer proteins, related to Figures 1, 2, and 5 (A) The prototype sequence is from Wuhan-1 reference strain. The mutation sites of the variants were indicated. SP, signal peptide; NTD, N-terminal domain; RBD, receptor-binding domain; TM, transmembrane domain; CTD, C-terminal domain. (B) Prototype RBD-dimer protein. (C) Beta RBD-dimer protein. (D) Prototype-Beta chimeric RBD-dimer protein. The gel filtration was carried out in HiLoad 16/600 Superdex 200 pg (A–C). (E) Delta-Omicron chimeric RBD-dimer protein. The gel filtration was carried out in Superdex 200 Increase 10/300 GL. The 280-nm absorbance curves are shown. SDS-PAGE migration was carried out in non-reduced and reduced conditions (B–E).

**Figure 1:**
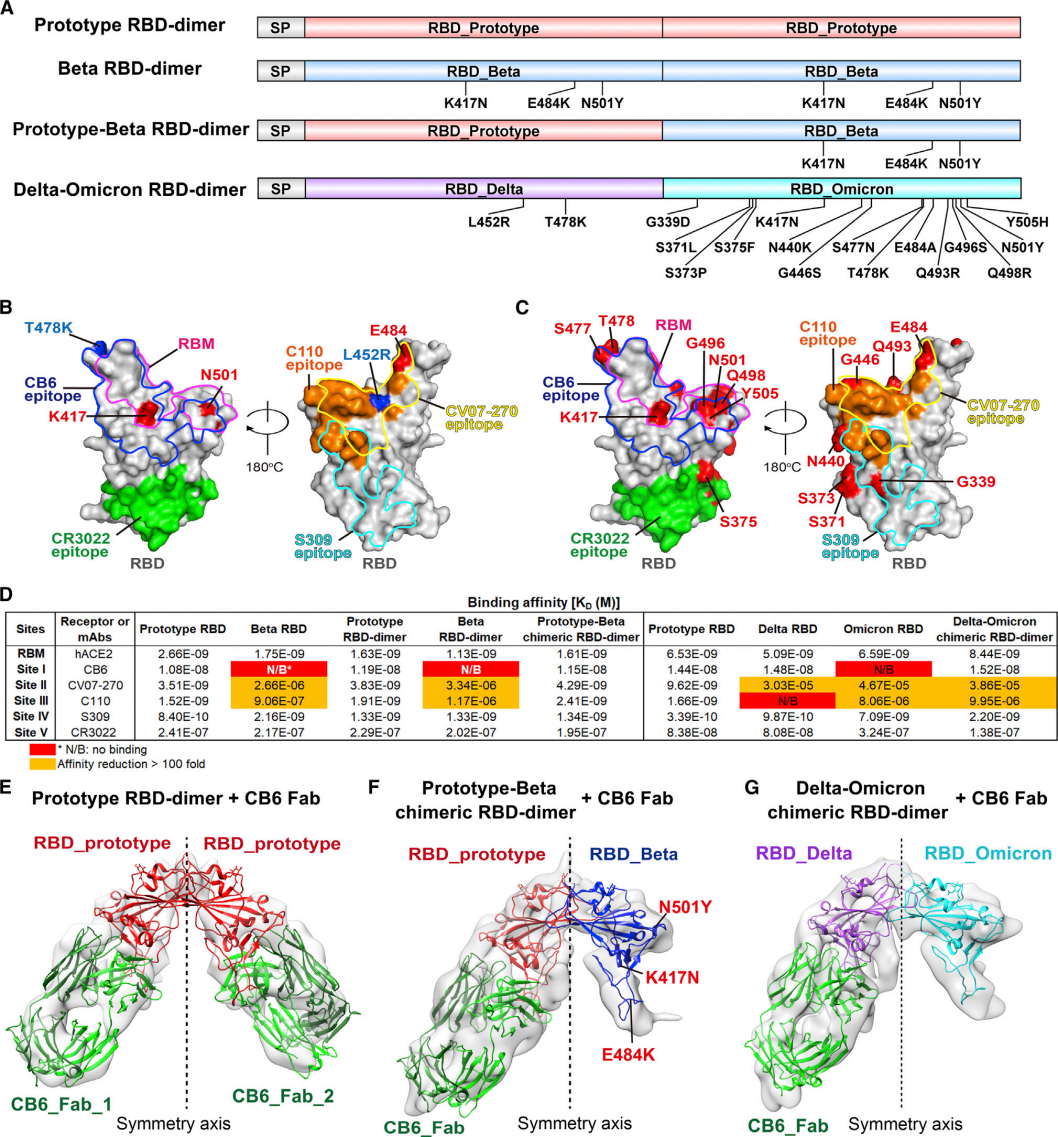
Antigenic and structural characterization of homotypic and chimeric RBD-dimers (A) Schematic diagram of prototype RBD-dimer, Beta RBD-dimer, prototype-Beta chimeric RBD-dimer, and Delta-Omicron chimeric RBD-dimer. Two SARS-CoV-2 RBDs were dimerized as tandem repeat (SP, signal peptide). (B) Footprint of hACE2 and five classes of antibodies on SARS-CoV-2 RBD. Prototype RBD is shown as gray surface (PDB: 6LZG). Residues that mutated in SARS-CoV-2 Beta and Delta variants are colored in red and blue, respectively. Footprints of hACE2 (PDB: 6LZG), CB6 (PDB: 7C01), CV07-270 (PDB: 6XKP), C110 (PDB: 7K8V), S309 (PDB: 6WPT), and CR3022 (PDB: 6W41) are highlighted in pink, blue, yellow, orange, cyan, and green, respectively. (C) Footprint of hACE2 and five classes of antibodies on SARS-CoV-2 RBD. Prototype RBD is shown as gray surface. Residues that mutated in SARS-CoV-2 Omicron variants are colored in red. (D) Binding affinities of antigens bound to hACE2 and representative mAbs targeting five major sites. Red indicates no binding (N/B). The ones with affinity reductions more than 100-fold are colored in yellow. (E) Density map of prototype RBD-dimer bound to two CB6 Fabs, with the atomic models of the SARS-CoV-2 RBD/CB6 complex (PDB:7C01) were fitted and rebuilt. (F) Density map of prototype-Beta chimeric RBD-dimer bound to one CB6 Fab, with the atomic models of SARS-CoV-2 RBD (PDB: 6LZG) and RBD/CB6 complex (PDB: 7C01) were fitted and rebuilt, clearly showing that Beta variant RBD does not bind to CB6. (G) Density map of Delta-Omicron chimeric RBD-dimer bound to one CB6 Fab, with the atomic models of Delta RBD (PDB: 7V8B), Omicron RBD (PDB: 7WBL) and CB6 (PDB: 7C01) were fitted and rebuilt, clearly showing that Omicron variant RBD does not bind to CB6.

**Figure S2:**
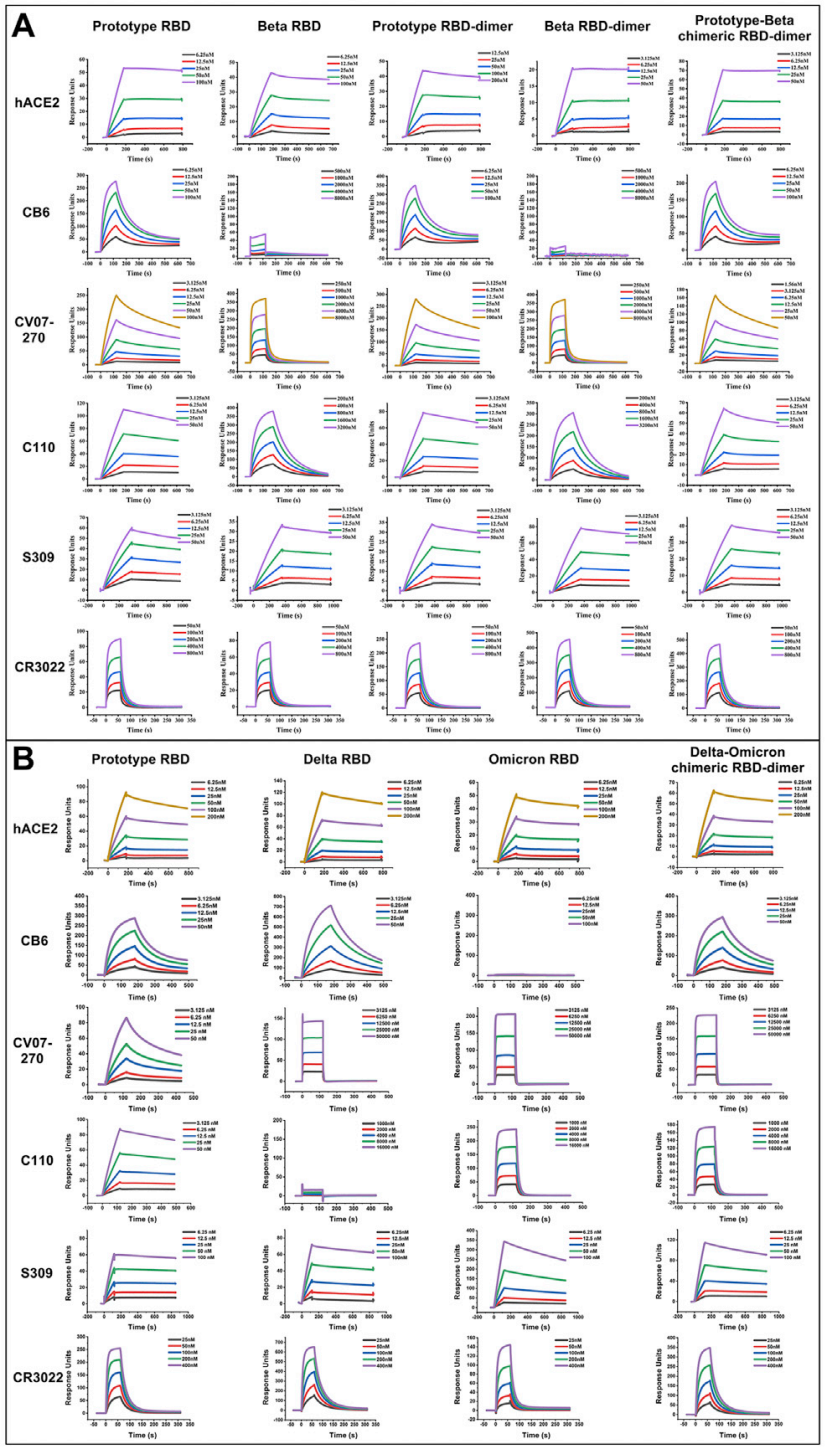
BIAcore diagram of RBD-monomer and RBD-dimer proteins bound to hACE2 and mAbs, related to Figure 1 (A) Prototype RBD, Beta RBD, prototype RBD-dimer, Beta RBD-dimer, or prototype-Beta chimeric RBD-dimer proteins bound to hACE2 and mAbs. (B) Prototype RBD, Delta RBD, Omicron RBD, or Delta-Omicron chimeric RBD-dimer proteins bound to hACE2 and mAbs. The antigen proteins were immobilized on the chip and were tested for binding with gradient concentrations of hACE or mAb Fabs as indicated using multi-cycle mode by BIAcore 8000. The binding profiles are shown with time (s) on the x axis and response units (RUs) on the y axis.

The chimeric RBD-dimer vaccine design strategy was first tested in the surge of Beta variant in early 2021. The RBD of Beta variant has three mutations: K417N, E484K, and N501Y (Figure S1A). To adapt Beta variant, we designed two immunogens based on the original prototype RBD-dimer: (1) two copies of Beta RBD and (2) the C-terminal prototype RBD linked N-terminal Beta RBD as a prototype-Beta chimera (Figure 1A). These immunogens were expressed in Expi293F cells and purified as single dimer-sized proteins (molecular weight ∼60 kDa) as verified by analytical gel filtration and gel electrophoresis (Figures S1B–S1D). Next, surface plasmon resonance (SPR) assay was performed to verify the exposure of both receptor-binding motif (RBM) and major antigenic sites, using receptor protein of human angiotensin converting enzyme (hACE2) (Wang et al., 2020) and SARS-CoV-2 RBD-specific mAbs as probes. Representative mAbs target five major antigenic sites with available structures (Figure 1B; Barnes et al., 2020; Kreye et al., 2020; Pinto et al., 2020; Shi et al., 2020; Yuan et al., 2020). Monomeric prototype and Beta RBD proteins were tested for comparison. As expected, all these immunogens showed similar binding affinities to hACE2 (apparent K_D_ ranging from 1.13 to 2.66 nM) (Figures 1D and S2A). Compared with the prototype RBD, both monomeric and dimeric forms of Beta RBD showed dramatically decreased binding affinities to mAb CB6 (no detectable binding), CV07-270 (>100-fold reduction), and C110 (>100-fold reduction), respectively, demonstrating the antigenic changes. By contrast, both prototype and prototype-Beta chimeric RBD-dimer preserved the binding activities to almost all tested mAbs (Figures 1D and S2A). This is because in the prototype-Beta chimeric design, the prototype domain can bind well with all these mAbs, including CB6.

### Cryoelectron microscopy (cryo-EM) structural analysis of RBD-dimers

**Figure S3:**
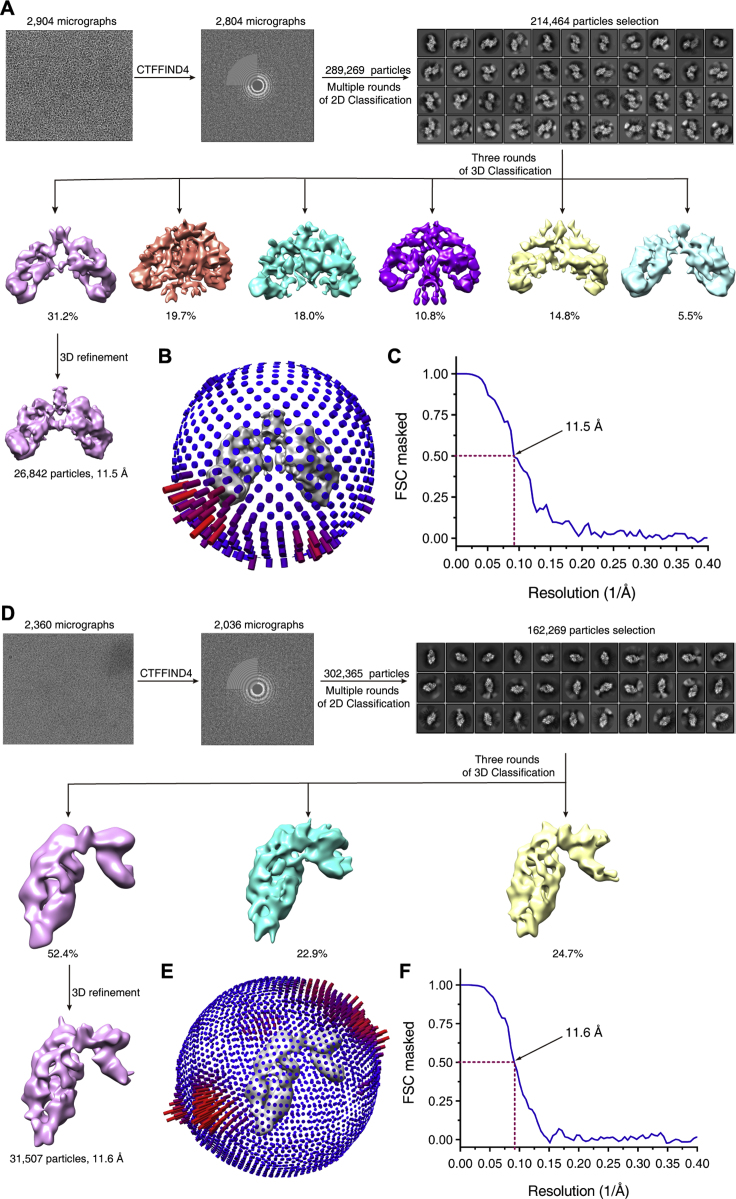
Cryo-EM analysis of CB6 Fab in complexed with prototype RBD-dimer and prototype-Beta chimeric RBD-dimer, respectively, related to Figure 1 (A–C) Cryo-EM analysis of CB6 Fab in complexed with prototype RBD-dimer. (D–F) Cryo-EM analysis of CB6 Fab in complexed with prototype-Beta chimeric RBD-dimer. (A and D) Flow chart of cryo-EM data processing for RBD-dimers bound to CB6. (B and E) Euler angle distribution of the final reconstruction. (C and F) The FSC curve for the reconstruction, the FSC 0.5 cutoff value is indicated by red dashed line.

**Figure S4:**
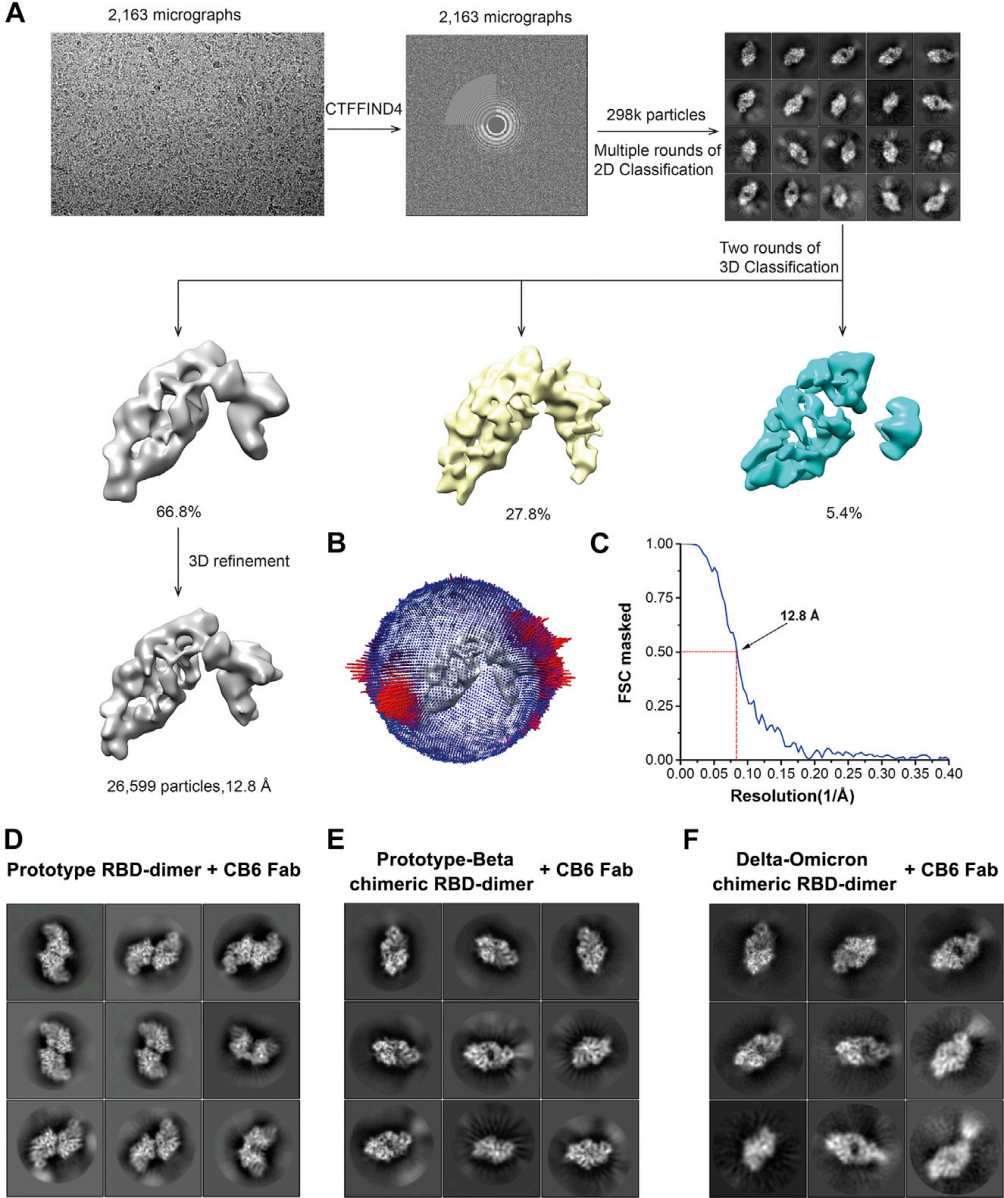
Cryo-EM analysis of CB6 Fab in complexed with Delta-Omicron chimeric RBD-dimer and representative cryo-EM 2D class average images of RBD-dimers bound to CB6 Fabs, related to Figure 1 (A) Flow chart of cryo-EM data processing for Delta-Omicron chimeric RBD-dimer bound to CB6. (B) Euler angle distribution of the final reconstruction. (C) The FSC curve for the reconstruction, the FSC 0.5 cutoff value is indicated by red dashed line. (D) Representative cryo-EM 2D class average images of prototype RBD-dimer bound to two CB6 Fabs. (E) Representative cryo-EM 2D class average images of prototype-Beta chimeric RBD-dimer bound to one CB6 Fab. (F) Representative cryo-EM 2D class average images of Delta-Omicron chimeric RBD-dimer bound to one CB6 Fab.

For further structural characterization, we determined the cryo-EM structures of fragment antigen binding (Fab) of CB6 in complex with either prototype or prototype-Beta chimeric RBD-dimer. Since the two copies of RBD tethered by the flexibly N and C termini, both the homotypic and chimeric RBD-dimers showed a variety of conformations, precluding the structure analysis at high resolution (Figures S3A–S3F). After image reconstruction by two-dimensional (2D) classification and 3D refinement, the prototype and prototype-Beta chimeric RBD-dimers bound to CB6 Fabs were determined to 11.5- and 11.6-Å resolution of mass density maps, respectively, into which the previously published atomic models of SARS-CoV-2 RBD and RBD/CB6 complex were fitted and rebuilt (Figures S3A–S3F). The rebuilt models showed their overall conformations. Like MERS-CoV RBD-dimer structure as we reported earlier (PDB: 7C02) (Dai et al., 2020), both prototype and prototype-Beta chimeric SARS-CoV-2 RBD-dimers arranged as “bilateral lung”-like structures with axial symmetry (Figures 1E, 1F, S4D, and S4E). Two RBD subunits stack together via core domains of each other with their external domains exposed. As expected, prototype RBD-dimer is in complexed with dual CB6 Fabs at both arms (Figure 1E). By contrast, prototype-Beta chimeric RBD-dimer only engages one CB6 Fab at one arm, which is consistent with the fact that Beta RBD with K417N mutation is resistant to CB6 binding (Figure 1F; Starr et al., 2021; Wang et al., 2021b). In summary, the antigenic characterization and structural analysis demonstrated that the recombinant RBD-dimers correctly presented RBM and major neutralizing epitopes.

### Immunogenicity and protection efficacy of prototype-Beta chimeric RBD-dimer vaccine in mice

**Figure 2:**
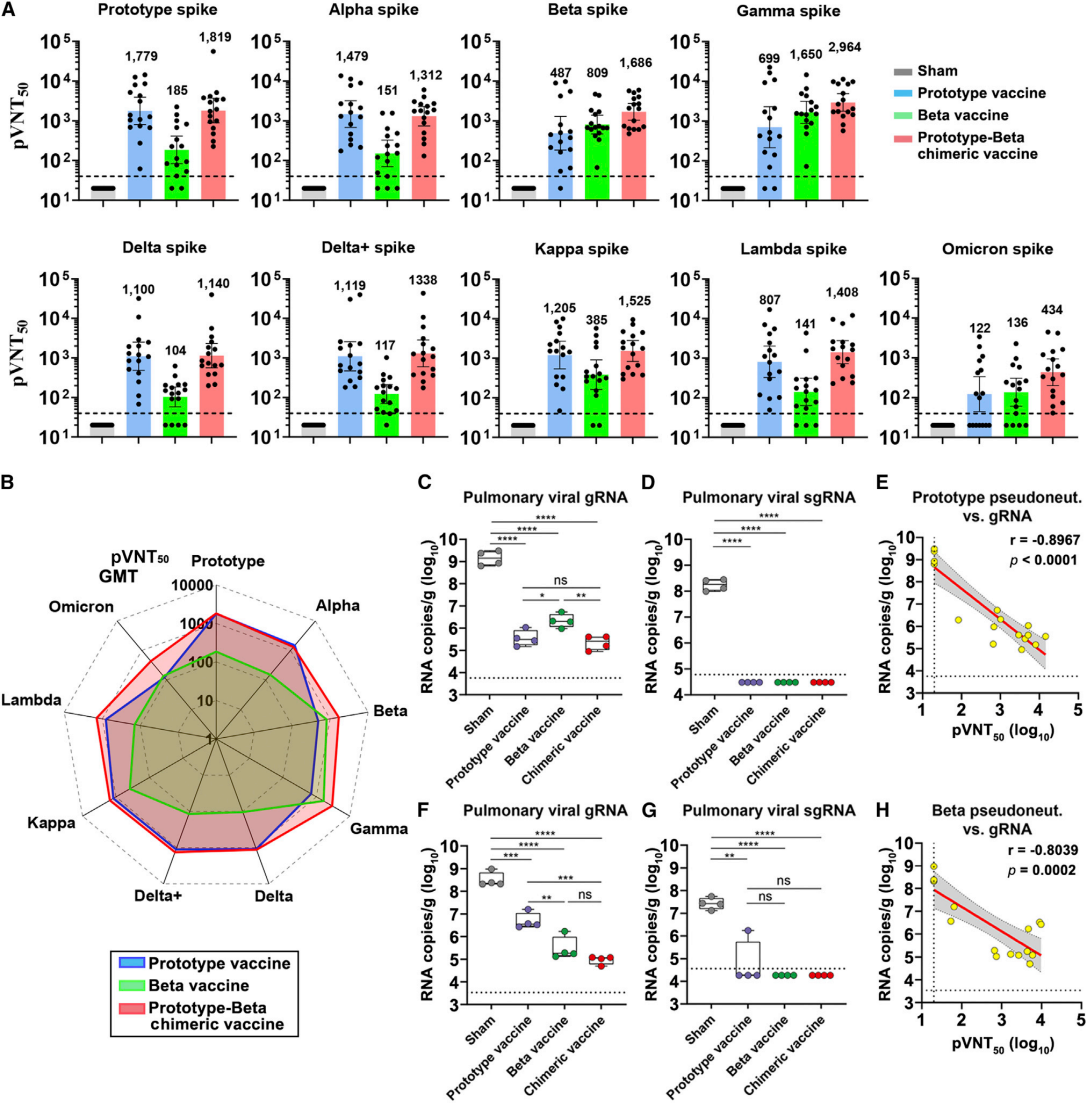
Immunogenicity and protection efficacy of homotypic and prototype-Beta chimeric RBD-dimers Two batches of 8- to 10-week-old female BALB/c mice (n = 8 each batch) were immunized with two doses of 0.5-μg prototype, Beta or prototype-Beta chimeric RBD-dimer using AddaVax as adjuvant, 21 days apart. PBS plus adjuvant was given as the sham control. The first batch of BALB/c mice (n = 8) receiving two doses of vaccine or sham were challenged with Beta variant or prototype SARS-CoV-2 via the i.n. route at 46 and 61 days, respectively, post the second vaccination. (A) Sera collected at 14 days post the second immunization were tested for neutralization of a panel of pseudotyped viruses displaying prototype, Alpha, Beta, Gamma, Delta, Delta plus, Kappa, Lambda, and Omicron spikes. The values are the GMT ± 95% confidence interval (CI). The horizontal dashed line indicates the lower limit of detection (LLOD). (B) Radar plot demonstrating the neutralization profile of sera elicited by prototype, Beta or prototype-Beta chimeric vaccine against eight SARS-CoV-2 pseudotyped viruses. (C–H) Random selection of four mice in each group were challenged with 5 × 10^5^ TCID_50_ of prototype SARS-CoV-2 (GISAID: EPI_ISL_514256-7) (C–E) and the other four were challenged with 1 × 10^6^ TCID_50_ of Beta variant (GDPCC-nCoV84 strain) (F–H). Mice challenged with prototype SARS-CoV-2 had received Ad5-hACE2 intranasally 5 days before. (C) Pulmonary viral gRNA levels were detected by qRT-PCR. (D) Pulmonary viral sgRNA levels were detected by qRT-PCR. (E) Plots show correlations and corresponding two-sided p values between pVNT_50_ of prototype SARS-CoV-2 and viral gRNA. (F) Pulmonary viral gRNA levels were detected by qRT-PCR. (G) Pulmonary viral sgRNA levels were detected by qRT-PCR. (H) Plots show correlations and corresponding two-sided p values between pVNT_50_ of Beta variant and viral gRNA. For (C, D, F, and G), shown are the box and whiskers plots of 25th to 75th percentile with median as center and whiskers of minimum to maximum percentile. p values were analyzed with two-tailed unpaired t test (ns, p > 0.05; ^∗^p < 0.05; ^∗∗^p < 0.01; ^∗∗∗^p < 0.001; ^∗∗∗∗^p < 0.0001). For (E and H), red and gray lines indicate linear regression line and 95% CI, respectively. r and p values represent Spearman’s correlation coefficients and corresponding two-sided p values, respectively. Symbols represent individual mouse and may overlap for equal values. Horizontal dashed lines indicated the LLOD.

**Figure S5:**
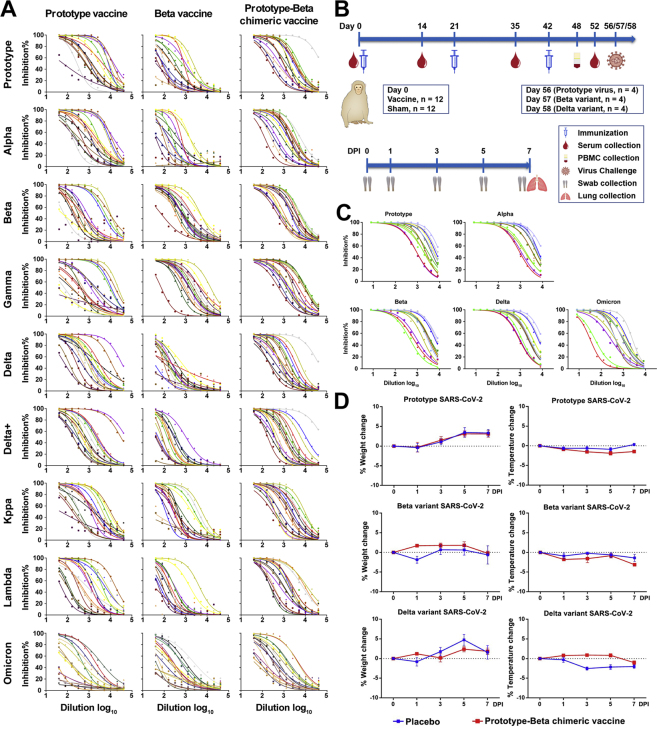
Evaluation of the immunogenicity and protection efficacy of prototype-Beta chimeric RBD-dimer vaccine in animals, related to Figures 2, 3, and 4 (A) Antisera from mice vaccinated with prototype, Beta or prototype-Beta chimeric RBD-dimer vaccines were tested neutralization of a panel of pseudotyped viruses displaying prototype, Alpha, Beta, Gamma, Delta, Delta plus, Kappa, Lambda and Omicron spike, respectively. (B) Rhesus monkeys (n = 12) were immunized with clinical-grade prototype-Beta chimeric vaccine (25 μg antigen + aluminum hydroxide adjuvant) or placebo (aluminum hydroxide adjuvant). Three doses were administrated on days 0, 21, and 42. These monkeys were bled for humoral immunogenicity evaluation before immunization and 14, 35, and 52 days after receiving the first dose. Blood was collected for cellular immune responses on day 48. At the days 56, 57, and 58, four prototype-Beta chimeric RBD-dimer-vaccinated macaques and four sham-vaccinated macaques were challenged with total 1 × 10^6^ TCID_50_ prototype SARS-CoV-2, Beta variant and Delta variant, respectively. Nasal swabs, throat swabs and anal swabs were collected before challenging and at the days 1, 3, 5, 7 post infection for SARS-CoV-2 titration. Lungs were collected for virus titration and pathological examination on 7 DPI. (C) Antisera from vaccinated macaques were tested neutralization of a panel of pseudotyped viruses displaying prototype, Alpha, Beta, Delta and Omicron spike, respectively. (D) The macaques were challenged with prototype virus, Beta variant and Delta variant, respectively, and weight and temperature were monitored. The values are means ± SEM.

To assess the vaccine immunogenicity, groups of BALB/c mice (two batches pooled together) were administrated with two doses of 0.5 μg prototype, Beta or prototype-Beta chimeric RBD-dimer, adjuvanted with AddaVax (a squalene-based adjuvant), 21 days apart. PBS plus adjuvant was given as sham control. Mouse serum samples were collected 14 days after the second dose. We used a panel of pseudotyped viruses (vesicular stomatitis virus backbone) displaying SARS-CoV-2 spikes to test the sera neutralizing activities against variants, including the prototype (Wuhan-Hu-1 strain), all five VOCs (Alpha [B.1.1.7], Beta [B.1.351], Gamma [P.1], Delta [B.1.617.2], Delta plus [B.1.617.2 with an additional K417N substitution in spike], and Omicron [B.1.1.529]) and two variants of interest (VOIs) (Kappa [B.1.617.1] and Lambda [C.37]) (Figure S1A). Two doses of prototype vaccine elicited high levels of NAbs against pseudovirus displaying prototype spike, with geometric mean titer (GMT) of 50% pseudovirus neutralization titer (pVNT_50_) up to 1,779 (Figures 2A and S5A). The pVNT_50_ GMT exhibited varying reductions against pseudovirus displaying variant spikes (Figures 2A and S5A). Antisera showed slight decline in neutralization of pseudovirus displaying Alpha spike (GMT, 1,479) but exhibited more pronounced reductions in neutralization of pseudoviruses displaying Beta (GMT, 487), Gamma (GMT, 699), Delta (GMT, 1,100), Delta plus (GMT, 1,119), Kappa (GMT, 1,205), Lambda (GMT, 807), and Omicron (GMT, 122) spikes (Figure 2A). Omicron variant contains the most mutations in RBD (15 sites) (Figure S1A) and is the most resistant to sera neutralization (14.6-fold reduction). Beta and Gamma variants, containing similar three mutations at RBD (K417N/T, E484K, and N501Y) (Figure S1A), were the other two with large (2.5- to 3.7-fold) reductions. On the contrary, Beta RBD-dimer vaccine induced enhanced neutralizing responses against pseudotyped viruses displaying Beta and Gamma spikes (GMT 809 and 1,650, respectively) (Figure 2A) but largely reduced responses against prototype and other variant pseudoviruses (GMTs ranging from 104 to 385) (Figure 2A). Encouragingly, prototype-Beta chimeric RBD-dimer vaccine elicited balanced antibody responses to neutralize Omicron (GMT 434) and all other pseudotyped viruses tested (GMTs ranging from 1,140 to 2,964). It outperformed prototype vaccine to elicit neutralizing responses against Beta, Gamma, and Omicron pseudotyped viruses (Figure 2A) and was also superior to the Beta vaccine to elicit antisera in neutralization of pseudotyped virus displaying prototype, Alpha, Delta, Delta plus, Kappa, Lambda, or Omicron spikes (Figure 2A). More intuitionally, the radar plot showed that prototype-Beta chimeric RBD-dimer vaccine induced the broadest and most balanced neutralizing activity against SARS-CoV-2 variants, compared with homotypic RBD-dimer vaccines (Figure 2B).

To further explore the protective efficacy of these RBD-dimers, the first batch of eight mice in each group were evaluated for protection against SARS-CoV-2 infection. BALB/c mouse is not sensitive to prototypic SARS-CoV-2 infection because of the low binding affinity between mouse ACE2 and S protein but becomes sensitive to Beta variant due to the promoted affinity by N501Y mutation at S protein (Niu et al., 2021). Therefore, four mice in each group were transduced via intranasal (i.n.) route with adenovirus (Ad5) expressing hACE2 and, 5 days later, challenged intranasally with prototypic SARS-CoV-2 (hCoV-19/China/CAS-B001/2020 strain). The other four mice in each group were challenged directly with Beta SARS-CoV-2 variant (GDPCC-nCoV84 strain) (Meng et al., 2021) via i.n. route. Mice were euthanized and necropsied at 5 days postinfection (DPI) to quantify viral genomic (g)RNA and subgenomic (sg)RNA, an indicator of viral replication (Wölfel et al., 2020), in lung. For mice challenged with prototype SARS-CoV-2, high levels of both viral gRNA (average: 1.72 × 10^9^ copies/g) and sgRNA (average: 1.9 × 10^8^ copies/g) were detected in sham-immunized mice (Figures 2C and 2D). By contrast, significantly reduced viral loads (p < 0.0001) were detected in vaccine-immunized mice. Averages of pulmonary viral gRNA were 4.61 × 10^5^, 2.58 × 10^6^, and 2.66 × 10^5^ copies/g in prototype, Beta and prototype-Beta chimeric vaccine groups, respectively, with 2–4-log_10_ reduction compared with the sham group (Figure 2C). In line with the trends in neutralization, both prototype and prototype-Beta chimeric vaccine groups showed significantly lower viral gRNA compared with the Beta vaccine group (p = 0.016 and 0.0045, respectively) (Figure 2C). All vaccine groups showed undetectable pulmonary viral sgRNA, indicating the complete control of viral replication (Figure 2D). Analysis of immune correlates of protection following vaccination showed that NAb titers correlated strongly with the reduction of pulmonary prototypic SARS-CoV-2 gRNA based on a linear model (r = −0.8967, p < 0.0001) (Figure 2E).

For mice challenged with SARS-CoV-2 Beta variant, high levels of pulmonary viral gRNA (average: 4.01 × 10^8^ copies/g) and sgRNA (average: 3.03 × 10^7^ copies/g) were detected in sham-vaccinated mice (Figures 2F and 2G). Less than 2-log_10_ viral reductions were observed in the prototype vaccine group (average gRNA: 6.34 × 10^6^ copies/g; average sgRNA: 4.51 × 10^5^ copies/g) (Figures 2F and 2G). In contrast, more significant reduction (2–4-log_10_) of pulmonary viral gRNA was detected in mice vaccinated with Beta (average: 5.46 × 10^5^ copies/g; p = 0.0079 compared with the prototype vaccine group) or prototype-Beta chimeric vaccine (average: 9.81 × 10^4^ copies/g; p = 0.0001 compared with the prototype vaccine group) (Figure 2F). No sgRNA can be detected in both groups, suggesting the completely control of viral replication (Figure 2G). In the context of Beta SARS-CoV-2 challenge, NAb titers and pulmonary viral gRNA were also inversely correlated with one another according to a linear model (r = −0.8039, p = 0.0002) (Figure 2H).

**Figure S6:**
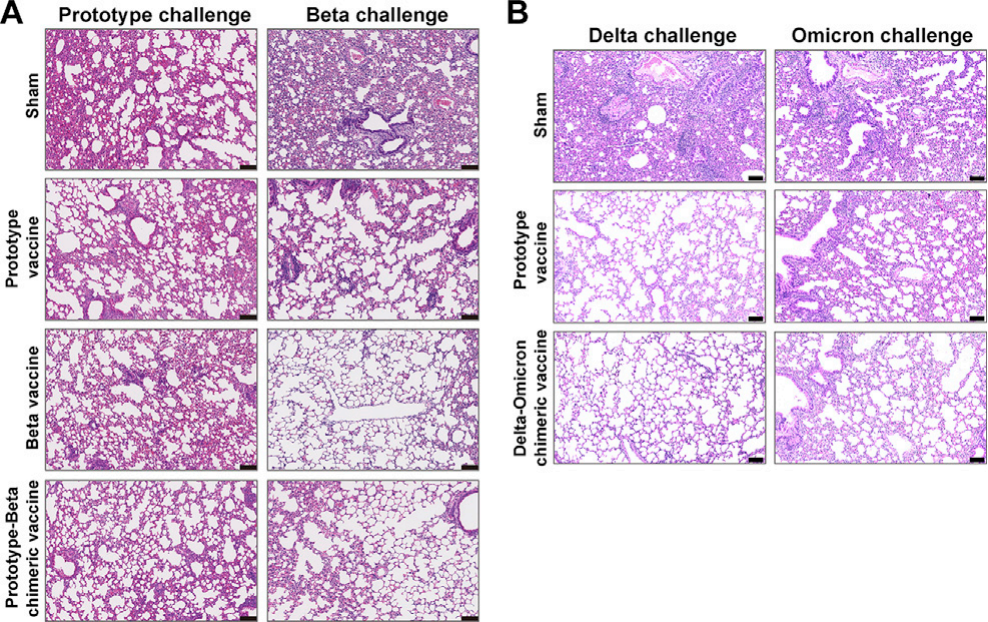
Histological pathology of lung sections of mice, related to Figures 2 and 5 (A) Shown are the typical lung sections from mice challenged with prototype SARS-CoV-2 or Beta variant by H&E staining. Scale bars, 100 μm. (B) Shown are the typical lung sections from mice challenged with SARS-CoV-2 Delta or Omicron variant by H&E staining. Scale bars, 100 μm.

To provide a further assessment of protection following vaccination, mice in each group were assessed for virus-related pathology in lung at 5 DPI. Tissue sections were stained with hematoxylin and eosin (H&E) to examine histopathology. Sham recipients challenged with either prototype virus or Beta variant showed moderate-to-severe histopathological changes in lung, including vanishment of alveolar cavities, pulmonary vascular congestion, and diffuse inflammatory cell infiltration (Figure S6A). In contrast, mice vaccinated with prototype, Beta or prototype-Beta chimeric RBD-dimer vaccine exhibited relieved lung injury (Figure S6A). Moreover, histopathology showed that both Beta and prototype-Beta chimeric vaccines provided better protection compared with prototype vaccine when mice were challenged with SARS-CoV-2 Beta variant (Figure S6A). The histopathology result was consistent with the tendency of pulmonary viral gRNA shown above and demonstrated the more balanced protection conferred by prototype-Beta chimeric RBD-dimer vaccine.

### Immunogenicity of prototype-Beta chimeric RBD-dimer vaccine in rhesus macaques

We next selected the prototype-Beta chimeric RBD-dimer immunogen for further preclinical study. Pilot scale immunogen protein was produced in clinical-grade CHO cell line and formulated with alum-based adjuvant as vaccine. Alum-only buffer was produced as the sham control.

**Figure 3:**
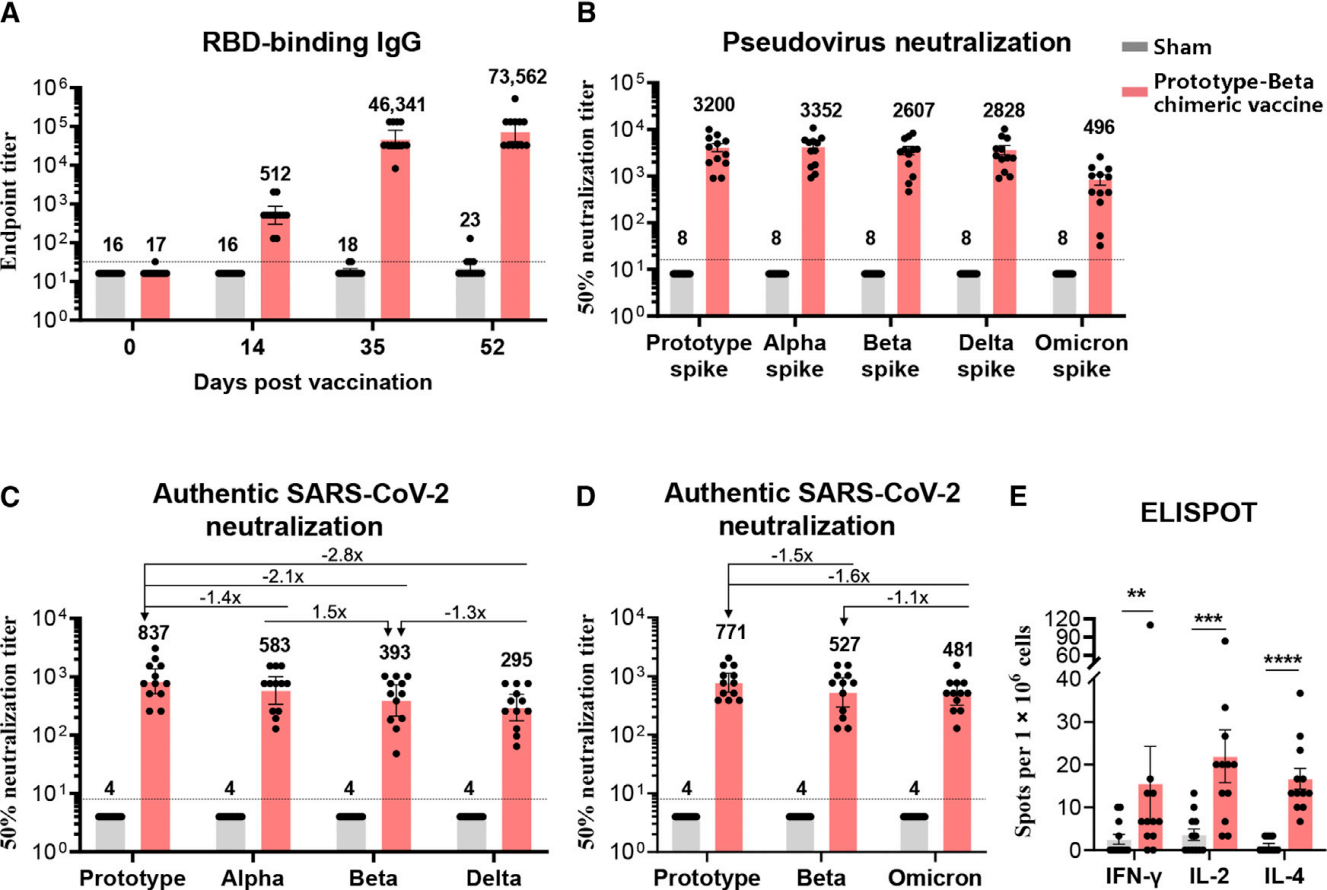
Immunogenicity of prototype-Beta chimeric vaccine in rhesus macaques Twenty-four rhesus macaques (see Table S1) were immunized with 3 doses of prototype-Beta chimeric RBD-dimer vaccine or sham (n = 12), 21 days apart. Serum samples were collected according to the study schedule shown in Figure S5B. (A) Endpoint titer of antigen-binding IgG. (B) 50% neutralization titer of pseudotyped virus (prototype, Alpha, Beta, Delta, and Omicron) in serum. (C) 50% neutralization titer of authentic SARS-CoV-2 (prototype, Alpha, Beta, and Delta). (D) 50% neutralization titer of authentic SARS-CoV-2 (prototype, Beta and Omicron). The values shown in (A)–(D) are the GMT ± 95% confidence interval (CI). (E) Summed IFN-γ, IL-2 and IL-4 ELISPOT responses in PBMCs collected at day 6 after the third dose toward peptides spanning SARS-CoV-2 RBD. The values are means ± SEM. p values were analyzed with two-tailed Mann-Whitney test. (^∗∗^p < 0.01; ^∗∗∗^p <0.001; ^∗∗∗∗^p < 0.0001).

Twenty-four healthy young rhesus macaques (Table S1) were immunized intramuscularly with three jabs of 25 μg vaccine (n = 12), a dose used in human (Yang et al., 2021), or sham (n = 12), 21 days apart. Serum samples were collected before (day 0) and after (days 14, 35, and 52) priming (Figure S5B). The average endpoint titers of RBD-binding antibody raised from 512 after one dose to 46,341 after two doses and further up to 73,562 after three doses in the vaccine group (Figure 3A). The sera after three doses were tested for neutralization of pseudotyped viruses expressing four VOCs (Figures 3B and S5C). Sera from vaccinated animals showed robust and balanced neutralizing activities against prototype, Alpha, Beta, and Delta pseudoviruses (GMT ranging between 2,607 and 3,200). All vaccine-elicited sera neutralized Omicron pseudovirus (GMT 496), with a 6.5-fold reduction of titer compared with prototype neutralization (Figure 3B). All vaccine-elicited sera neutralized authentic prototype SARS-CoV-2 (GMT 837), variant Alpha (GMT 583), Beta (GMT 393), and Delta (GMT 295) (Figure 3C). As the authentic Omicron variant (BA.1 subvariant, National Pathogen Resource Center of China (NPRC): 2.192100005 strain) was available in Chinese Center for Disease Control and Prevention (China CDC) currently, macaque sera were tested for neutralization of Omicron and showed 1.6-fold reduction of activity against Omicron than against prototype SARS-CoV-2 (Figure 3D). In addition, peripheral blood mononuclear cells (PBMCs) collected at day 6 after the third dose were tested for cytokine production using enzyme-linked immunospot (ELISPOT) assay. In consistent with trends of prototype RBD-dimer vaccine ZF2001 in rhesus macaque and human (An et al., 2022; Yang et al., 2021), prototype-Beta chimeric vaccine elicited moderate but balanced T_H_1 (IFN-γ and IL-2) and T_H_2 (IL-4) cytokine production (Figure 3E).

### Protective efficacy of prototype-Beta chimeric RBD-dimer vaccine in rhesus macaques

The 12 rhesus macaques receiving prototype-Beta chimeric vaccine were challenged (n = 4) with prototype SARS-CoV-2 (GDPCC-nCoV27 strain), Beta variant (GDPCC-nCoV84 strain), and Delta (CCPM-B-V-049-2105-8), respectively, via both the upper and lower respiratory tracts. The other 12 sham-immunized animals were also challenged in parallel as controls (n = 4) (Figure S5B). Body weight and temperature were monitored daily until euthanasia at 7 DPI (Figure S5D). Swabs were collected at 0, 1, 3, 5, and 7 DPI from nose, throat, and anus for viral gRNA quantification. At 7 DPI, lung tissues were collected from 7 lopes for histopathology examination. Lung tissues from each lope (a sample mixture of six sites) were quantified for viral gRNA.

**Figure 4:**
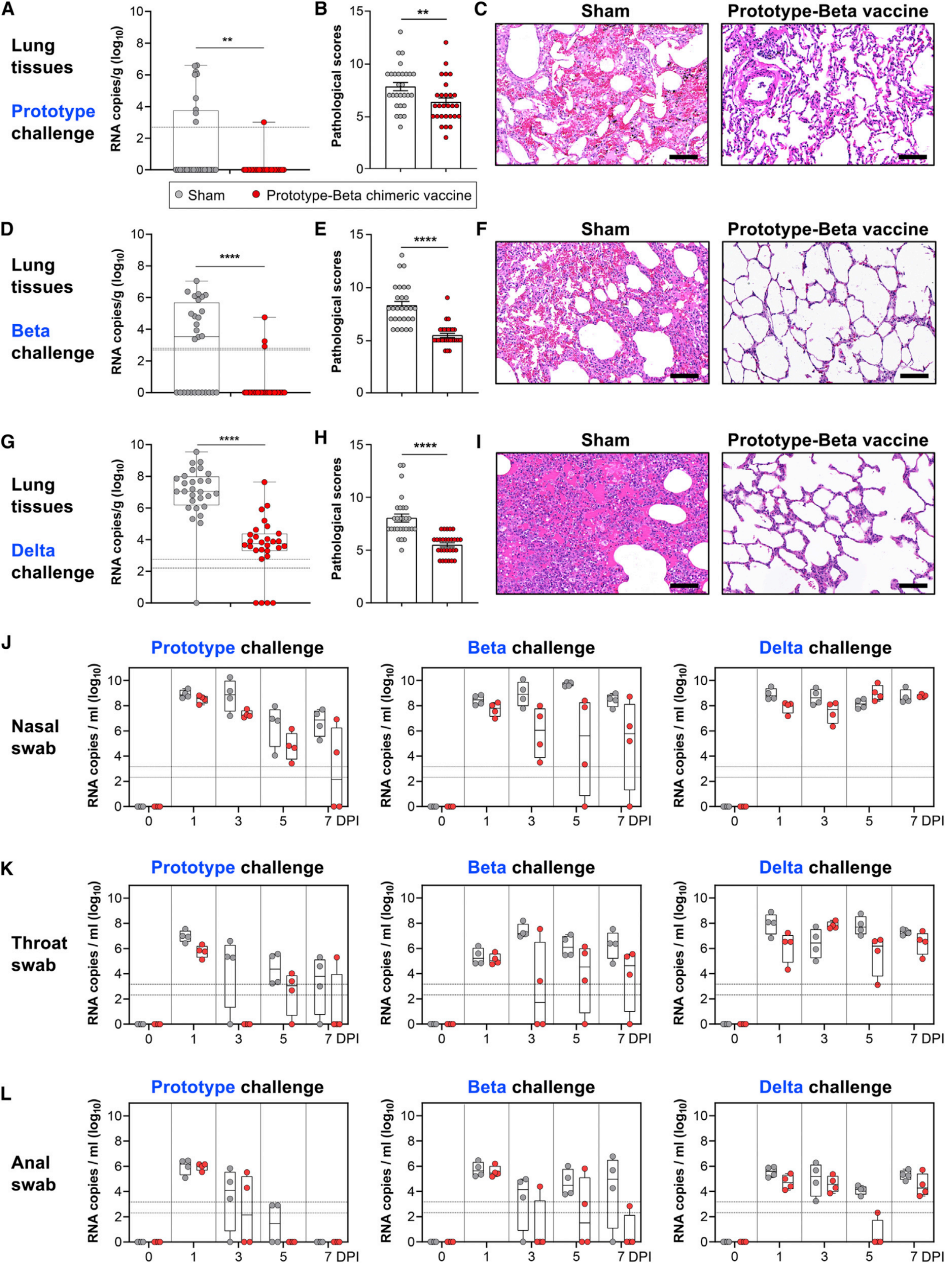
Protection of rhesus macaques by prototype-Beta vaccine immunization (A, D, and G) Box and whiskers plots of 25th–75th percentile with median as center and whiskers of minimum to maximum percentile prototype (A), Beta variant (D), and Delta variant (G) viral gRNA in lung tissues at 7 DPI. Each dot represents a lung lope. The 28 lopes from four animals in each group were analyzed together. p values were analyzed with two-tailed Mann-Whitney test (^∗∗^p < 0.01; ^∗∗∗∗^p < 0.0001). (B, E, and H) Pooled analyses of pathological scores for lung tissues of macaques challenged with prototype SARS-CoV-2 (B), Beta (E), and Delta (H). The values are means ± SEM. p values were analyzed with two-tailed Mann-Whitney test (^∗∗^p < 0.01; ^∗∗∗∗^p < 0.0001). (C, F, and I) Histological pathology analyses of lung sections of macaques challenged with prototype SARS-CoV-2 (C), Beta (F), and Delta (I). Black bar represents 100 μm. (J–L) Box and whiskers plots of 25th–75th percentile with median as center and whiskers of minimum to maximum percentile viral gRNA at 0, 1, 3, 5, and 7 DPI in nasal swabs (J), throat swabs (K), and anal swab (L). Each dot represents an animal sample. The horizontal dash lines represent limit of detection, with the upper and lower ones for highest and lowest limit of detection, respectively.

Viral loads (gRNA) were detected in many lung lopes of control animals, with the average 1.66-log_10_ copies per gram in prototype challenge group, 2.93-log_10_ in Beta challenge group, and 6.95-log_10_ in Delta challenge group (Figures 4A, 4D, and 4G). In contrast, significantly lower viral loads were detected in vaccinated animals, with only 1 and 3 lopes having detectable viral gRNA in prototype and Beta challenge group, respectively (Figures 4A and 4D). In comparison to sham-immunized macaques, lower viral loads were also detected in both upper respiratory tract (nasal and throat swabs) and anus (anal swab) of the vaccinated animals in all days after infections with prototype or Beta SARS-CoV-2 (Figures 4J–4L). However, the vaccine protection against Delta viral gRNA in upper airway is less pronounced (Figures 4J and 4K).

At 7 days post inoculation of prototype, Beta or Delta SARS-CoV-2, all sham-immunized macaques developed moderate-to-severe pneumonia, with thickened alveolar septa, vanishment of pulmonary alveolar, congestion, and massive inflammatory cell infiltration in alveoli (Figures 4B, 4C, 4E, 4F, 4H, and 4I). In contrast, none of vaccinated animals developed severe pneumonia, with the pathological scores significantly lower than that of the sham group (Figures 4B, 4C, 4E, 4F, 4H, and 4I). Histopathology examination demonstrated that prototype-Beta chimeric RBD-dimer vaccine can dramatically relieve the lung injury by either infection of prototype, Beta or Delta SARS-CoV-2.

### Development of Delta-Omicron chimeric RBD-dimer vaccine

In the late 2021, Omicron variant were circulating globally and gradually substituted its predecessor Delta (Karim and Karim, 2021). The mutations in the spike protein of Omicron variant lead to the resistance for humoral immune responses induced by early SARS-CoV-2 infection and vaccination (Cameroni et al., 2022; Dejnirattisai et al., 2022; Hoffmann et al., 2022). In the context of cocirculation of Delta and Omicron variants, we developed the Delta-Omicron vaccine with our chimeric RBD-dimer approach (Figure 1A).

The Delta-Omicron chimeric RBD-dimer protein was expressed and purified with high yield and purity, indicating the capacity for large-scale production (Figure S1E). Next, the exposure of RBM and major antigenic sites of RBD-dimer vaccine were verified through SPR assay using hACE2 protein and major classes of RBD-specific mAbs as probes (Figures 1C and 1D). Monomeric RBD proteins from prototype, Delta, and Omicron variants were used for comparison. Delta-Omicron chimeric RBD-dimer showed similar binding affinity (8.44 nM) to hACE2 in comparison with the affinities of monomeric prototype (6.53 nM), Delta (5.09 nM), and Omicron RBD (6.59 nM) (Figures 1D and S2B). For the mAb binding, the Delta RBD did not bind to mAb C110 and exhibited decreased binding affinity to mAb CV07-270; the Omicron RBD did not bind to mAb CB6 and displayed reduced binding affinities to mAbs CV07-270 and C110 (Figures 1D and S2B). In contrast, Delta-Omicron chimeric RBD-dimer bound all the tested representative mAbs, but like a combination of Delta and Omicron RBD with the decreased affinities to mAbs CV07-270 and C110, respectively (Figures 1D and S2B).

The cryo-EM structure of Delta-Omicron chimeric RBD-dimer in complexed with CB6 Fab was determined at resolution of 12.8 Å. It reassembles the structure of prototype-Beta chimeric RBD-dimer in complexed with CB6 Fab as the “bilateral lung”-like structure engaging one CB6 Fab at the Delta arm (Figures 1G, S4A–S4C, and S4F). In summary, the antigenic characterization and structural analysis indicated that the Delta-Omicron chimeric RBD-dimer protein was correctly folded, presenting the RBM and major antigenic epitopes.

### Immunogenicity and protection efficacy of Delta-Omicron chimeric vaccine in mice

**Figure 5:**
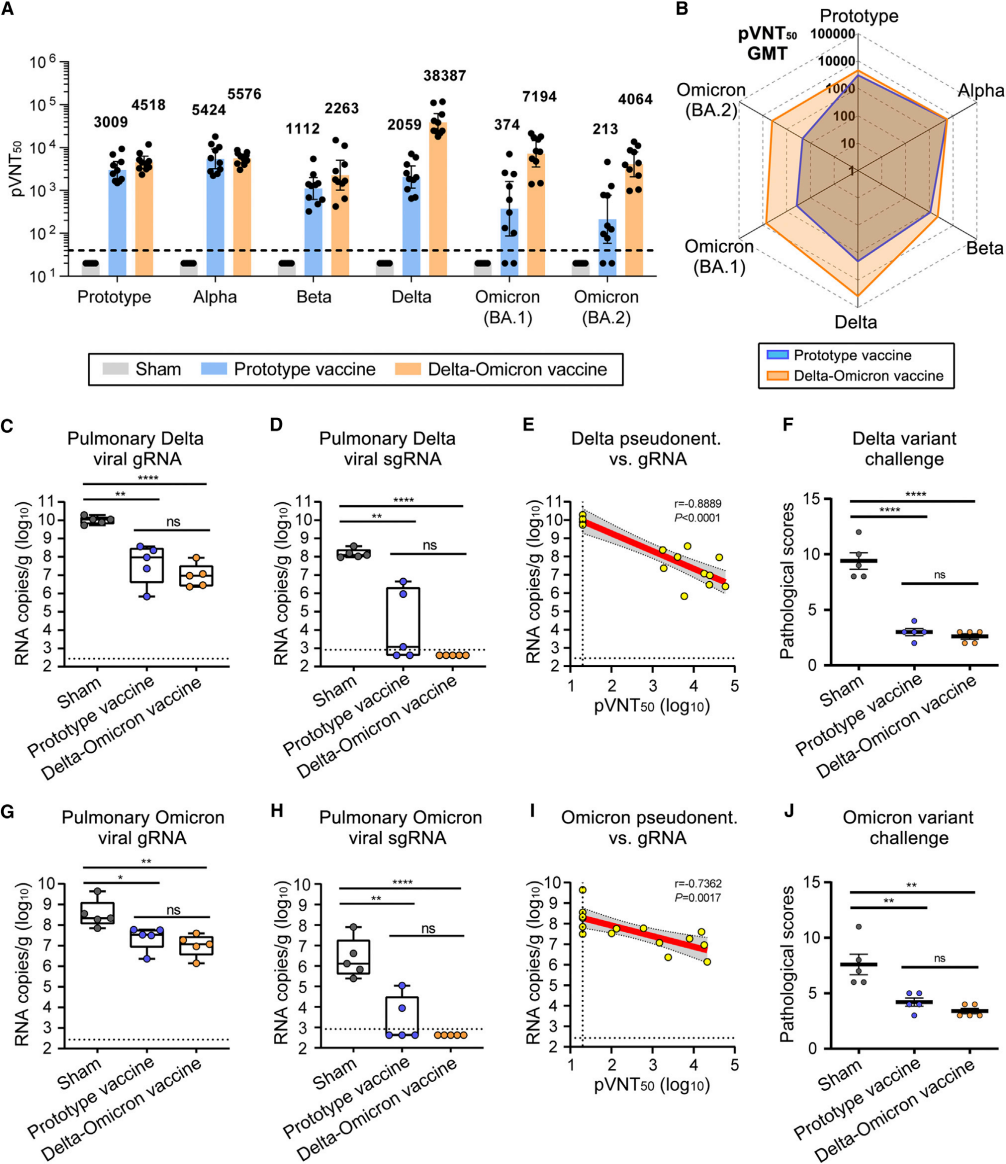
Immunogenicity and protection efficacy of Delta-Omicron chimeric RBD-dimer vaccine Groups of 6- to 8-week-old female BALB/c mice (n = 10) were immunized with two doses of 2-μg prototype or Delta-Omicron chimeric RBD-dimer using AddaVax as adjuvant, 21 days apart. PBS plus adjuvant was given as the sham control. Sera collected at 14 days post the second immunization for antibody titration. (A) Mice sera were tested for neutralization of a panel of pseudotyped viruses displaying prototype, Alpha, Beta, Delta, Omicron (BA.1), and Omicron (BA.2) spike. The values are the GMT ± 95% confidence interval (CI). The horizontal dashed line indicates the lower limit of detection (LLOD). (B) Radar plot demonstrating the neutralization profile of sera elicited by prototype vaccine or Delta-Omicron chimeric vaccine against five SARS-CoV-2 pseudotyped viruses. (C–J) Random selection of five mice in each group were challenged with 6 × 10^5^ TCID_50_ of Delta SARS-CoV-2 variant (CCPM-B-V-049-2105-8) (C–F) and the other five were challenged with 6 × 10^5^ TCID_50_ of Omicron (BA.1) variant (CCPM-B-V-049-2112-18) at 58 days post the second immunization (G–J). Mice challenged with Delta variant had received Ad5-hACE2 intranasally 5 days before. (C) Pulmonary Delta viral gRNA levels were detected by qRT-PCR. (D) Pulmonary Delta viral sgRNA levels were detected by qRT-PCR. (E) Plots show correlations and corresponding two-sided p values between pVNT_50_ of Delta variant and Delta viral gRNA. (F) Analyses of pathological scores for lung tissues of mice challenged with Delta variant. (G) Pulmonary Omicron viral gRNA levels were detected by qRT-PCR. (H) Pulmonary Omicron viral sgRNA levels were detected by qRT-PCR. (I) Plots show correlations and corresponding two-sided p values between pVNT_50_ of Omicron variant and Omicron viral gRNA. (J) Analyses of pathological scores for lung tissues of mice challenged with Omicron variant. For (C, D, G, and H), shown are the box and whiskers plots of 25th–75th percentile with median as center and whiskers of minimum to maximum percentile. p values were analyzed with two-tailed unpaired t test (ns, p > 0.05; ^∗^p < 0.05; ^∗∗^p < 0.01; ^∗∗∗∗^p < 0.0001). For (E and I), red and gray lines indicate linear regression line and 95% CI, respectively. r and p values represent Spearman’s correlation coefficients and corresponding two-sided p values, respectively. Symbols represent individual mouse and may overlap for equal values. Horizontal dashed lines indicated the LLOD. For (F and J), the values are means ± SEM. p values were analyzed with two-tailed Mann-Whitney test (ns, p > 0.05; ^∗∗^p < 0.01; ^∗∗∗∗^p < 0.0001).

To evaluate the immunogenicity of Delta-Omicron chimeric vaccine, BALB/c mice were immunized with two doses of AddaVax adjuvanted Delta-Omicron chimeric protein or homotypic prototype RBD-dimer protein, 21 days apart. Delta-Omicron vaccine elicited high titers of serological NAbs against pseudotyped virus displaying Delta, Omicron (BA.1), and Omicron (BA.2) spike proteins, respectively, with 18.6, 19.2, and 19.1 times of those induced by homotypic prototype RBD-dimer vaccine (Figure 5A). Meanwhile, the GMTs of neutralizing activities against prototype, Alpha, and Beta SARS-CoV-2 pseudovirus were all higher in the Delta-Omicron vaccine group in comparison with the homotypic prototype vaccine group, demonstrating the outperformance of Delta-Omicron chimeric vaccine to induce broader immune responses (Figures 5A and 5B).

To evaluate the protection efficacy of Delta-Omicron chimeric vaccine, the immunized BALB/c mice (n = 10) were randomly divided into two batches (n = 5) and challenged with SARS-CoV-2 Delta (CCPM-B-V-049-2105-8 strain) and Omicron (BA.1 subvariant, CCPM-B-V-049-2112-18 strain) variants, respectively. Since wild-type BALB/c mice were sensitive to Omicron variant but not Delta variant, the mice for Delta variant challenge were transduced with Ad5 expressing hACE2 5 days before virus inoculation. At 3 DPI, all mice were euthanized and necropsied, and lung samples were collected for virus titration.

For mice challenged with Delta variant, the averages of pulmonary viral gRNA were 1.09 × 10^10^ in the sham group but reduced to 1.43 × 10^8^ copies/g in the prototype vaccine group, and additionally reduced (474-fold) to 2.37 × 10^7^ copies/g in the Delta-Omicron chimeric vaccine group (Figure 5C). In line with this, the pulmonary viral sgRNA were detected in all mice in the sham group with high levels (average: 1.70 × 10^8^ copies/g), but only detectable in three mice receiving prototype vaccine with the average titer of 1.07 × 10^6^ copies/g and undetectable in the all mice receiving Delta-Omicron vaccine, suggesting the complete control of Delta viral replication (Figure 5D).

For mice challenged with Omicron variant, the sham group of mice developed high levels of pulmonary viral gRNA (average: 1.04 × 10^9^ copies/g) and sgRNA (average: 1.73 × 10^7^ copies/g) (Figures 5G and 5H). Mice immunized prototype vaccine showed decreased pulmonary viral gRNA (average: 3.68 × 10^7^ copies/g) and sgRNA (2.41 × 10^4^ copies/g) (Figures 5G and 5H). Encouragingly, the protection efficacy of Delta-Omicron vaccine was better than prototype vaccine, with no pulmonary virus replication detected in all mice (Figure 5H). NAb titers inversely correlated with the reduction of pulmonary gRNA of both Delta variant (r = −0.8889, p < 0.0001) and Omicron variant (r = −0.7362, p < 0.0017) based on a linear model (Figures 5E and 5I).

The histopathological analysis showed that at 3 days post either Delta or Omicron variant infection, sham-immunized mice developed pulmonary damage, including inflammatory cell infiltration, congestion, and vanishment of alveolar cavities (Figure S6B). As a comparison, both prototype and Delta-Omicron chimeric vaccine-immunized mice showed alleviative lung injury, with the pathological scores significantly lower than those of the sham group (Figures 5F and 5J). Delta-Omicron chimeric vaccine showed lower pathological scores in comparison with the homotypic prototype form, indicating a better protection (Figures 5G, 5H, and 5J).

## Discussion

SARS-CoV-2 variants have caused waves of new infections worldwide, including the recent Omicron outbreak. To fight against the SARS-CoV-2 variants, we developed a chimeric RBD-dimer vaccine approach, which induced broader immune responses than homotypic RBD-dimer vaccines. Based on this approach, we firstly designed the prototype-Beta chimeric RBD-dimer vaccine and then the Delta-Omicron chimeric vaccine.

In the first half of 2021, Beta variant was highly concerned because of its greatly decreased sensitivity to vaccine-elicited sera and its reduction of vaccine efficacy in clinical trials and effectiveness in the real world (Abu-Raddad et al., 2021; Chemaitelly et al., 2021; Garcia-Beltran et al., 2021; Hoffmann et al., 2021a; Li et al., 2020; Lucas et al., 2021; Madhi et al., 2021; Shinde et al., 2021; Tang et al., 2021; Tegally et al., 2021; Xu et al., 2021). Therefore, we present a chimeric vaccine design using RBD-dimer to cover both prototype and Beta variants. The prototype-Beta vaccine induced broader NAbs against SARS-CoV-2 variants than its antigenically homotypic counterparts, especially, with higher activities to cross-neutralize the recently circulating Omicron variant pseudotyped virus (Figure 2A). Accordingly, prototype-Beta chimeric version showed better protection against SARS-CoV-2, compared with both homotypic prototype and Beta vaccines. The nonhuman primates study showed that high viral loads of Delta SARS-CoV-2 were detected in both upper respiratory tract and lung of all macaques. Our data supported the finding that Delta variant caused frequent breakthrough infections in the real world (Levine-Tiefenbrun et al., 2021; Singanayagam et al., 2022). Although Delta variant was more difficult to be cleared in the upper airway, prototype-Beta chimeric RBD-dimer vaccine significantly decreased viral loads of 3.4-log_10_ in lung and prevented virus-induced lung lesion (Figures 4G–4I).

In the second half of 2021, Delta and Omicron VOCs emerged one after another and became the circulating variants successively. The Omicron variant carries mutations at as many as 32 positions in the S protein, including 15 positions in the RBD, and has showed strongly reduced sensitivity to the approved vaccine-elicited sera (Cameroni et al., 2022; Cao et al., 2022; Cele et al., 2022; Dejnirattisai et al., 2022; Hoffmann et al., 2022; Liu et al., 2022; Planas et al., 2022; VanBlargan et al., 2022). In the present study, we also observed that pseudotyped virus displaying Omicron spike was the most resistant to prototype vaccine-elicited antisera (14.6-fold reduction in neutralization) (Figure 2A). However, the sera elicited by prototype-Beta vaccine showed 3.6-fold higher than that induced by homotypic prototype vaccine (Figure 2A). Recently, in the context of the global cocirculation of Delta and Omicron variants, we rapidly developed the Delta-Omicron vaccine with our chimeric RBD-dimer approach. The Delta-Omicron vaccine outcompeted the homotypic prototype vaccine to prevent infections by either Delta or Omicron variants in mice and was also balanced to counter prototype and the other VOCs. Interestingly, recent studies showed the Omicron-specific mRNA vaccine induced mice sera that potently neutralized Omicron variant but showed limited neutralization of historical D614G or other SARS-CoV-2 variants, such as Beta and Delta variants (Lee et al., 2022; Ying et al., 2022). Consistently, WHO advised to develop multivalent vaccines as next generation of COVID-19 vaccine candidates for induction of broader immune responses against both circulating and emerging variants (WHO, 2022). Our result supported the variant-adapted multivalent vaccines are superior to homotypic prototype or variant-specific vaccine in the context of the fast-changing variant circulation.

Previous studies described the use of the ancestral SARS-CoV-2 RBD scaffolded immunogens to induce robust NAbs against ancestral SARS-CoV-2 and, concomitantly, enhance the broadly reactive NAbs against VOCs in monkeys; however, the neutralizing titers elicited against VOCs declined when compared with that against the ancestral SARS-CoV-2 (Arunachalam et al., 2021; Saunders et al., 2021). In contrast, we used an alternative approach, heterotypic RBD-dimer connected in tandem, to induce broad and balanced neutralizing responses against both ancestral SARS-CoV-2 and variants. In view of the approval of homotypic RBD-dimer-based vaccine ZF2001 and the high efficacy of this vaccine in Phase 3 clinical trials (Dai et al., 2022), heterotypic RBD-dimer chimera designs would be a feasible approach to rapidly adapt SARS-CoV-2 variants or even other coronaviruses in future.

In the structural analysis of RBD-dimer antigens, both prototype-Beta and Delta-Omicron chimeric RBD-dimers are observed to be arranged as similar “bilateral lung”-like structures, exposing five major antigenic sites. This supports the versatility of the tandem RBD-dimer as the module to adapt different coronaviruses for the induction of broader immune responses. The present study was timely and encouraging for our fight against COVID-19 pandemic, especially for the Omicron rampage. The Delta-Omicron chimeric vaccine is under development by Anhui Zhifei Longcom Biopharmaceutical Co., Ltd. This design can also be applied in other vaccine platforms, such as mRNA, DNA, and viral vectors.

### Limitations of the study

First, the immunogenicity and protection efficacy of Delta-Omicron chimeric vaccine was not evaluated in the nonhuman primate model because of the limited animal supply at this moment. We are trying to arrange it. Second, the current vaccines used in humans are based on the ancestral prototype strain, and new variant-specific vaccines should be considered for their role as boosters as well as first-time immunizations. Our data demonstrated the variant-adapted multivalent vaccines were superior to the monovalent prototype- or variant-matched vaccine as first-time immunizations. However, it would be important to further explore whether these vaccines also outperform the others as boosters, given more than 10 billion doses of prototype-based COVID-19 vaccines have been administrated globally.

## STAR★Methods

### Key resources table

**Table:** 

REAGENT or RESOURCE	SOURCE	IDENTIFIER
**Antibodies**		

Goat Anti-Monkey IgG H&L (HRP)	Abcam	Cat#ab112767; RRID:AB_10866625
CB6	Shi et al., 2020	N/A
CV07-270	Kreye et al., 2020	N/A
C110	Barnes et al., 2020	N/A
S309	Pinto et al., 2020	N/A
CR3022	Yuan et al., 2020	N/A

**Bacterial and virus strains**		

Prototype SARS-CoV-2, hCoV-19/China/CAS-B001/2020 strain	Isolated from a SARS-CoV-2 infected patient by IMCAS, China	GISAID: EPI_ISL_514256-7
Prototype SARS-CoV-2, IVDC-QD-11-2P2 strain	Isolated from a SARS-CoV-2 infected patient by China CDC	N/A
Prototype SARS-CoV-2, GDPCC-nCoV27 strain	Imported from Guangdong CDC, China	N/A
Alpha variant SARS-CoV-2, SARS-CoV-2/C-Tan-BJ202101(B1.1.7) strain	Isolated from a SARS-CoV-2 infected patient by China CDC	NPRC: 2. 062100002
Beta variant SARS-CoV-2, GDPCC-nCoV84 strain	Imported from Guangdong CDC, China	NPRC: 2.062100001
Delta variant SARS-CoV-2, CCPM-B-V-049-2105-8 strain	Imported from Chongqing CDC, China	N/A
Omicron (BA.1) variant SARS-CoV-2	Isolated from a SARS-CoV-2 infected patient by China CDC	NPRC: 2.192100005
Omicron (BA.1) variant SARS-CoV-2, CCPM-B-V-049-2112-18 strain	Imported from Institute of Laboratory Animals Science, CAMS & PUMC, China	N/A
SARS-CoV-2 pseudovirus	This paper	N/A
Ad5-hACE2	Provided by Gary Wong, Institut Pasteur of Shanghai, CAS, China	N/A

**Biological samples**		

Serum samples from BALB/c mice	This paper	N/A

Serum samples from rhesus macaques	This paper	N/A
**Chemicals, peptides, and recombinant proteins**		

AddaVax adjuvant	InvivoGen	Cat#vac-adx-10
Prototype SARS-CoV-2 RBD peptide pool	Beijing SciLight Biotechnology Ltd. Co.	N/A
Recombinant prototype SARS-CoV-2-S protein RBD monomer, spike residues 319-541, GenBank: YP_009724390	Dai et al., 2020	N/A
Recombinant Beta variant SARS-CoV-2-S protein RBD monomer, spike residues 319-541, GISAID: EPI_ISL_736940	This paper	N/A
Recombinant Delta variant SARS-CoV-2-S protein RBD monomer, spike residues 319-541, GenBank: OK091006.1	This paper	N/A
Recombinant Omicron (BA.1) variant SARS-CoV-2-S protein RBD monomer, spike residues 316-534, GISAID: EPI_ISL_6795848	ACROBiosystems	Cat#SPD-C522e
Recombinant prototype SARS-CoV-2-S protein RBD-dimer, spike residues 319-537, two copies in tandem, GenBank: YP_009724390	Dai et al., 2020	N/A
Recombinant Beta variant SARS-CoV-2-S protein RBD-dimer, spike residues 319-537, two copies in tandem, GISAID: EPI_ISL_736940	This paper	N/A
Recombinant prototype-Beta chimeric RBD-dimer, spike residues 319-537 (prototype, GenBank: YP_009724390) and 319-537 (Beta variant, GISAID: EPI_ISL_736940)	This paper	N/A
GMP grade of recombinant prototype-Beta chimeric RBD-dimer, spike residues 319-537 (prototype, GenBank: YP_009724390) and 320-537 (Beta variant, GISAID: EPI_ISL_736940)	Anhui Zhifei Longcom Biopharmaceutical Co. Ltd	N/A
Recombinant Delta-Omicon chimeric RBD-dimer, spike residues 319-537 (Delta variant, GenBank: OK091006.1) and 316-534 (Omicron variant, GISAID: EPI_ISL_6795848)	This paper	N/A
Recombinant hACE2 protein, residues 1-740	Sino Biological Inc.	Cat#10108-H08H

**Critical commercial assays**		

HisTrap HP 5 mL column	GE Healthcare	Cat#17524802
HiLoad™ 16/600 Superdex™ 200 pg column	GE Healthcare	Cat#28989335
Superdex™ 200 Increase 10/300 GL column	GE Healthcare	Cat#28990944
HiTrap Protein A HP	Cytiva	Cat#17040303
Series S Sensor Chip CM5	GE Healthcare	Cat# 29-1496-03
Trizol	Thermo Fisher Scientific	Cat# 10296028
FastKing One Step Probe RT-qPCR kit	Tiangen Biotech	Cat#FP314
TaqMan Fast Virus 1-Step Master Mix kit	Thermo Fisher Scientific	Cat#4444434
Monkey INF-γ ELISpot^PLUS^ kit (ALP)	MabTech	Cat#3421M-4APW-10
Monkey IL-2 ELISpot^PLUS^ kit (ALP)	MabTech	Cat#3445M-4APW-10
Human IL-4 ELISpot^PRO^ kit (ALP)	MabTech	Cat#3410-2APW-10

**Deposited data**		

Cryo-EM structure of SARS-CoV-2 prototype RBD-dimer bound to CB6 Fab	This paper	EMDB:EMD-33225
Cryo-EM structure of SARS-CoV-2 prototype-Beta chimeric RBD-dimer bound to CB6 Fab	This paper	EMDB:EMD-33234
Cryo-EM structure of SARS-CoV-2 Delta-Omicron chimeric RBD-dimer bound to CB6 Fab	This paper	EMDB:EMD-33235

**Experimental models: Cell lines**		

Expi293F Cells	Thermo Fisher Scientific	Cat#A14527; RRID:CVCL_D615
Vero cells	ATCC	CCL-81; RRID:CVCL_0059
Vero-E6 cells	ATCC	CRL-1586; RRID:CVCL_0574
CHOZN® CHO K1 host cell line	SAFC	Cat#CHOK1-1VL

**Experimental models: Organisms/strains**		

BALB/c mice	Beijing Vital River Laboratory Animal Technology Co., Ltd. (licensed by Charles River)	N/A
Rhesus macaques	Qingwei Macaque Breeding Centre, Chengkou County, Chongqing, China	N/A

**Oligonucleotides**		

gRNA-F, GACCCCAAAATCAGCGAAAT	GENEWIZ and Thermo Fisher Scientific	N/A
gRNA-R, TCTGGTTACTGCCAGTTGAATCTG	GENEWIZ and Thermo Fisher Scientific	N/A
gRNA-probe, ACCCCGCATTACGTTTGGTGGACC	GENEWIZ and Thermo Fisher Scientific	N/A
sgRNA-F, CGATCTCTTGTAGATCTGTTCTC	GENEWIZ and Thermo Fisher Scientific	N/A
sgRNA-R, ATATTGCAGCAGTACGCACACA	GENEWIZ and Thermo Fisher Scientific	N/A
sgRNA-probe, ACACTAGCCATCCTTACTGCGCTTCG	GENEWIZ and Thermo Fisher Scientific	N/A
gRNA-Omicron-probe, ACTCCGCATTACGTTTGGTGGACC	Thermo Fisher Scientific	N/A

**Recombinant DNA**		

pCAGGS	MiaoLingPlasmid	N/A
pCAGGS_prototype_SARS-CoV-2_RBD_monomer, residues 319-541, GenBank: YP_009724390	Dai et al., 2020	N/A
pCAGGS_Beta_variant_SARS-CoV-2_RBD_monomer, residues 319-541, GISAID: EPI_ISL_736940	This paper	N/A
pCAGGS_Delta_variant_SARS-CoV-2_RBD monomer, residues 319-541, GenBank: OK091006.1	This paper	N/A
pCAGGS_prototype_SARS-CoV-2_RBD-dimer, residues 319-537, two copies in tandem, GenBank: YP_009724390	Dai et al., 2020	N/A
pCAGGS_Beta_variant_SARS-CoV-2_RBD-dimer, residues 319-537, two copies in tandem, GISAID: EPI_ISL_736940	This paper	N/A
pCAGGS_prototype-Beta_chimeric_RBD-dimer, residues 319-537 (prototype, GenBank: YP_009724390) and 319-537 (Beta variant, GISAID: EPI_ISL_736940)	This paper	N/A
pCAGGS_Delta-Omicon_chimeric_RBD-dimer, residues 319-537 (Delta variant, GenBank: OK091006.1) and 316-534 (Omicron variant, GISAID: EPI_ISL_6795848)	This paper	N/A
pCAGGS_CB6 Fab light chain	This paper	N/A
pCAGGS_CB6 Fab heavy chain	This paper	N/A
pCAGGS_CV07-270 light chain	This paper	N/A
pCAGGS_CV07-270 heavy chain	This paper	N/A
pCAGGS_C110 light chain	This paper	N/A
pCAGGS_C110 heavy chain	This paper	N/A
pCAGGS_S309 light chain	This paper	N/A
pCAGGS_S309 heavy chain	This paper	N/A
pCAGGS_CR3022 light chain	This paper	N/A
pCAGGS_CR3022 heavy chain	This paper	N/A

**Software and algorithms**		

MotionCor2	Zheng et al., 2017	https://emcore.ucsf.edu/ucsf-motioncor2
CTFFIND4.1	Rohou and Grigorieff, 2015	N/A
RELION3.1	Zivanov et al., 2018	http://www2.mrc-lmb.cam.ac.uk/relion
Chimera	Pettersen et al., 2004	http://www.cgl.ucsf.edu/chimera
CryoSPARC	Punjani et al., 2017	https://cryosparc.com/
BIAevaluation Version 3.0	GE Healthcare	N/A
GraphPad Prism	GraphPad Software	https://www.graphpad.com/
OriginPro 2018	OriginLab Cororation	http://www.OriginLab.com

**Other**		

BIAcore 8000	GE Healthcare	N/A
Vitrobot Mark IV	Thermo Fisher Scientifific	N/A
Quantifoil R 1.2/1.3 holey carbon grids	Quantifoil	N/A
Titan Krios microscope	Thermo Fisher Scientifific	N/A

### Resource availability

#### Lead contact

Further information and requests for resources and reagents should be directed to and will be fulfilled by the lead contact, George F. Gao (gaof@im.ac.cn).

#### Materials availability

All requests for unique/stable reagents generated in this study should be directed to and will be fulfilled by the lead contact author with a completed Materials Transfer Agreement.

### Experimental model and subject details

#### Cells and viruses

African green monkey kidney epithelial cells (Vero cells) (ATCC CCL81) and Vero E6 cells (ATCC CRL-1586) was maintained in Dulbecco’s modified Eagle’s medium (DMEM, Invitrogen, USA) supplemented with 10% fetal bovine serum (FBS) at 37°C under 5% CO_2_. Expi293F™ cells (Thermo Fisher Scientific) were cultured in medium at 37°C under 5% CO_2_. CHOZN® CHO K1 cell line was authenticated and the other cell lines were not. All cell lines were tested negative for mycoplasma contamination.

For experiments conducted in Institute of Microbiology, Chinese Academy of Science (IMCAS), prototype SARS-CoV-2 (hCoV-19/China/CAS-B001/2020, GISAID: EPI_ISL_514256-7) was propagated in Vero E6 cells and titrated by tissue culture infectious dose 50 (TCID_50_) assay on Vero E6 cells. For experiments conducted in Chinese Center for Disease Control and Prevention (China CDC), prototype SARS-CoV-2 (IVDC-QD-11-2P2), Beta variant (GDPCC-nCoV84, NPRC: 2.062100001) (Meng et al., 2021) and Omicron variant (BA.1, NPRC: 2.192100005) was propagated in Vero cells and titrated by TCID_50_ assay on Vero cells. For experiments conducted in Institute of Medical Biology, Chinese Academy of Medical Sciences (IMBCAMS), prototype SARS-CoV-2 (GDPCC-nCoV27), Alpha variant (NPRC: 2. 062100002), Beta variant (GDPCC-nCoV84, NPRC: 2.062100001), Delta variant (CCPM-B-V-049-2105-8) and Omicron variant (BA.1, CCPM-B-V-049-2112-18) were propagated in Vero E6 cells and titrated by TCID_50_ assay on Vero E6 cells.

#### Animals

Specific pathogen-free (SPF) female BALB/c mice were purchased from Beijing Vital River Laboratory Animal Technology Co., Ltd. (licensed by Charles River). All mice were allowed free access to water and standard chow diet and provided with a 12-hour light and dark cycle (temperature: 20-25°C, humidity: 40%-70%). All mice used in this study are in good health and are not involved in other experimental procedure. They were housed under SPF conditions in the laboratory animal facilities at IMCAS, China CDC and IMBCAMS. Mice were housed with 6 companions per cage. The challenge studies with prototype SARS-CoV-2 and Beta variant were conducted under animal biosafety level 3 (ABSL3) facility in IMCAS and China CDC, respectively. The mice experiments conducted in IMCAS were approved by the Committee on the Ethics of Animal Experiments of the IMCAS, and performed in compliance with the recommendations in the Guide for the Care and Use of Laboratory Animals of the IMCAS Ethics Committee. The challenge studies with SARS-CoV-2 Beta variant were approved by the Ethics Committee of the National Institute for Viral Disease Control and Prevention, China CDC. The challenge experiments with SARS-CoV-2 Delta and Omicron (BA.1) variants were approved by the Animal Ethics Committee of the IMBCAMS according to the National Guidelines on Animal Work in China. The age of mice at the time that experiments were performed were indicated in the corresponding figure legends.

Twelve female and twelve male 2–3 years-old rhesus macaques were purchased from Qingwei Macaque Breeding Centre, Chengkou County, Chongqing, China. All macaques are in good health and are not involved in other experimental procedure. These macaques were firstly housed in the laboratory animal facilities in Chongqing Medleader Bio-Pharm and immunized with vaccine candidate or sham. The age information of macaques was listed in the Table S1. The study schedule was shown in Figure S5B. All macaques were allowed free access to water and standard diet and provided with a 12-hour light and dark cycle (temperature: 21.1-23.7°C, humidity: 46.7%-63.3%). These macaques were following transferred to IMBCAMS and challenged with SRAS-CoV-2. The challenge experiments and authentic SARS-CoV-2 neutralizing antibody titration assays were performed with approval under Biosafety Level 3 (BSL3) and ABSL3 conditions by the Institutional Biosafety Committee of IMBCAMS in the Kunming National High-level Biosafety Primate Research Center. The challenge experiments on macaques were conducted under prior approval from the Animal Ethics Committee of the IMBCAMS according to the National Guidelines on Animal Work in China.

### Method details

#### Protein expression and purification

Monomeric prototype SARS-CoV-2 (GenBank: YP_009724390), Beta variant (GISAID: EPI_ISL_736940) and Delta variant (GenBank: OK091006.1) RBD contained S protein 319-541. Prototype SARS-CoV-2 RBD-dimer was two RBD (GenBank: YP_009724390, S protein residues 319–537) connected as tandem repeat. Beta SARS-CoV-2 RBD-dimer was two RBD (GISAID: EPI_ISL_736940, S protein residues 319–537) connected as tandem repeat. Prototype-Beta chimeric SARS-CoV-2 RBD-dimer was one prototype RBD (S protein residues 319–537) and one Beta RBD (S protein residues 319–537) connected as tandem repeat. Delta-Omicron chimeric SARS-CoV-2 RBD-dimer was one Delta RBD (S protein residues 319–537) and one Omicron BA.1 RBD (S protein residues 316–534) connected as tandem repeat.

For each construct, signal peptide sequence of MERS-CoV S protein (S protein residues 1-17) was added to the protein N terminus for protein secretion, and a hexa-His tag was added to the C terminus to facilitate further purification processes. These constructs were codon-optimized for mammalian cell expression and synthesized by GENEWIZ, China. These constructs were cloned into the pCAGGS vector, respectively, and transiently transfected into Expi293F™ cells. After 5 days, the supernatant was collected and soluble protein was purified by Ni affinity chromatography using a HisTrap™ HP 5 mL column (GE Healthcare). The samples were further purified via gel filtration chromatography with HiLoad™ 16/600 Superdex™ 200 pg (GE Healthcare) or Superdex™ 200 Increase 10/300 GL (GE Healthcare) in a buffer composed of 20 mM Tris-HCl (pH 8.0) and 150 mM NaCl. The eluted peaks were analyzed by SDS-PAGE for protein size and purity.

To explore the preclinical and clinical trials, GMP grade of prototype-Beta chimeric SARS-CoV-2 RBD-dimer protein was produced in CHO-K1 (SAFC) cell lines and purified by Anhui Zhifei Longcom Biopharmaceutical Co.Ltd, China. The prototype-Beta chimeric RBD-dimer was one prototype RBD (S protein residues R319-K537) and one Beta RBD (S protein residues V320-K537) connected as tandem repeat. The R319 was deleted in the RBD-dimer protein to avoid the potential protease cleavage. Signal peptide sequence was added to the N terminus for protein secretion. No tag sequence was added at C terminus.

The human monoclonal antibodies (mAbs) CV07-270, C110, S309 and CR3022 were transiently expressed in Expi293F™ cells. The cells were transfected with pCAGGS plasmids containing coding sequences for immunoglobulin heavy chain and light chain, and collected at days 5. The supernatant was mixed with one volume of buffer PBS, and filtered with a 0.22-μm filter. The mixture was passed through the HiTrap™ Protein A HP column (Cytiva). The bound protein was detached from the column by 0.1 M glycine, pH 3.0. The elution was adjusted to neutral pH by adding 1 M Tris-HCl, pH 9.0 and further purified by gel filtration. The antibody was finally buffered with PBS, concentrated and stored at −80 °C. To generated the fragment of antibody-binding (Fab), purified CV07-270, C110, S309 and CR3022 antibodies were digested with immobilized papain (Thermo Scientific) according to the manufacturer’s instructions. Fab fractions were purified by HiTrap™ Protein A HP column (Cytiva). To obtain mAb CB6 Fab, Expi293F™ cells were transfected with pCAGGS plasmids coding CB6 Fab, which was purified from the culture supernatant with HisTrap™ HP 5 mL column and HiLoad™ 16/600 Superdex™ 200 pg column in PBS buffer.

The monomeric Omicron (BA.1) variant RBD (S protein 316–534, GISAID: EPI_ISL_6795848) was purchased from ACROBiosystems, which was expressed from HEK293 cells.

The hACE2 protein (residues 1-740, Genbank: NP_068576) was purchased from Sino Biological Inc., China, which was expressed from HEK293T cells.

#### Surface plasmon resonance (SPR) assay

SPR binding experiments were carried out using a BIAcore 8000 device (GE Healthcare) at 25 °C. The buffers for all proteins used for kinetic analyses were exchanged to PBST (10 mM Na_2_HPO_4_; 2 mM KH_2_PO_4_, pH 7.4; 137 mM NaCl; 2.7 mM KCl; 0.005% Tween 20). Purified RBD-dimer and -monomer proteins were immobilized on a CM5 chip with the standard EDC/NHS coupling method at about 1,000 response units (RU). Serial dilutions of Fabs were prepared and used to flow over the chip surface. Data were collected over time. After each cycle, the sensor surface was regenerated via a short treatment using 10 mM NaOH. The apparent equilibrium dissociation constants (apparent binding affinity, K_D_) for each antibody were calculated using BIAcore 8000 analysis software (BIAevaluation v3.0). Each set of equilibrium binding responses was fitted to the 1:1 binding model.

#### Cryo-EM data collection and 3D reconstruction

For samples of prototype RBD-dimer bound to CB6 Fab and prototype-Beta chimeric RBD-dimer bound to CB6 Fab, an aliquot of 3.5 μl solution (0.4 mg/ml) was applied to glow-discharged Quantifiol R 1.2/1.3 holey carbon grids and blotted for 1.5 s with a humidity of 95% before being plunged into liquid ethane using a Vitrobot Mark IV (Thermo Fisher). The frozen grides were loaded onto a Titan Krios cryo-transmission electron microscope (Thermo Fisher) that is equipped with a BioQuantum energy filter (Gatan), operated at 300 kV for data collection. Automatic data collection was performed using Serial-EM software. Movies were recorded with a Gatan K2 direct electron in a super-resolution counting mode at pixel size of 0.68 Å. The exposure was performed with a dose rate of 15 e^**-**^/pixel/s and an accumulative dose of ∼60 e^**-**^/Å^2^ for each movie which was fractionated into 32 sub-frames. The final defocus range of the datasets was approximately-(1.2-3.0) μm.

The drift correction of all stacks were performed with MotionCor2 (Zheng et al., 2017) to generate 2 × binned images. Initial contrast transfer function (CTF) values for each micrograph were calculated with CTFFIND4.1 (Rohou and Grigorieff, 2015). Micrographs with an estimated resolution limit worse than 6.0 Å were discarded in the initial screening. The subsequent image processing and reconstruction were performed using Relion-3.1 (Zivanov et al., 2018) and cryoSPARC (Punjani et al., 2017).

For the prototype-Beta chimeric RBD-dimer/CB6 Fab dataset, 302,365 particles were picked from 2036 micrographs. Then the picked particles were extracted and subjected to two rounds of reference-free 2D classification in Relion. A clean dataset with 162,269 particles from good 2D classes were selected and the initial model was generated by cryoSPARC ab initio. Then the model was used as reference in Relion 3D classification. After the third round of 3D classification without applying symmetry, the predominant class containing a subset of 31,507 good particles. These particles were subjected to 3D refinement, which yielded a reconstruction at 11.6 Å resolution as determined by the Fourier shell correlation (FSC) 0.5 cut-off value.

The prototype RBD-dimer/CB6 Fab dataset was processed similarly. Briefly, a total of 289,269 automatically picked particles were extracted in Relion for the following 2D and 3D classification. Three rounds of reference-free 2D classification were performed to remove the heterogeneous particles. A clean dataset with 214,464 particles from good 2D classes was selected and subjected to three rounds of 3D classification without applying symmetry. A single dominant class (26,842 particles) was identified and used to calculate the density map at 11.5 Å resolution by applying a 3D refinement with C2 symmetry.

The Delta-Omicron chimeric RBD-dimer/CB6 Fab dataset was processed similarly to those described above. The specific images processing and reconstruction were shown in Figures S4A–S4C.

Due to the fierce flexibility between two RBDs, we could only obtain low resolution maps as described above. However, we fitted the crystal structure of RBD/CB6 complex (PDB: 7C01) or its RBD part into the two density maps using CHIMERA (Pettersen et al., 2004), which showed a high degree of matching.

#### Pseudotyped virus neutralization assay

The pseudotyped viruses displaying SARS-CoV-2 spikes express GFP in infected cells. They were prepared as previously described (Zhao et al., 2021). Mice sera were 2-fold serially diluted and incubated with pseudotyped virus at 37°C for 1 h. Then the mixture was transferred to pre-plated Vero cell monolayers in 96-well plates. After incubation for 15 h, the transducing unit numbers were calculated on a CQ1 confocal image cytometer (Yokogawa). Fifty percent pseudovirus neutralization titer (pVNT_50_) was determined by fitting nonlinear regression curves using GraphPad Prism and calculating the reciprocal of the serum dilution required for 50% neutralization of infection. pVNT_50_ below the limit of detection was determined as half the limit of detection.

#### Live SARS-CoV-2 neutralization assay

Neutralizing antibody activities induced by vaccines in rhesus macaques against prototype, Alpha, Beta and Delta SARS-CoV-2 were titrated on basis of inhibition of cytopathogenic effect (CPE) in IMBCAMS. Briefly, equal volume of serially diluted serum and 100 TCID_50_ SARS-CoV-2 was mixed and incubated for one hour at 37°C. The mixture of serum and virus was added to Vero E6 cells, followed by incubation at 37°C for 3 days. CPE was recorded for determination of antibody neutralizing titer. Prototype SARS-CoV-2 (GDPCC-nCoV27), Alpha (NPRC: 2.062100002), Beta (GDPCC-nCoV84, NPRC: 2.062100001) and Delta (CCPM-B-V-049-2105-8) variants were used in this study.

Neutralizing antibody activities induced by vaccines in rhesus macaques against Omicron variant were titrated on basis of CPE in China CDC. Titers of serological neutralizing antibodies against prototype and Beta SARS-CoV-2 were also measured for comparison. Briefly, equal volume of serially diluted serum and 100 TCID_50_ SARS-CoV-2 was mixed and incubated for one hour at 37°C. The mixture of serum and virus was added to Vero cells, followed by incubation at 37°C for 4 days. CPE was recorded for determination of antibody neutralizing titer. Prototype SARS-CoV-2 (IVDC-QD-11-2P2), Beta (GDPCC-nCoV84, NPRC: 2.062100001) and Omicron (BA.1, NPRC: 2.192100005) variants were used in this study.

#### Enzyme-linked immunosorbent assay (ELISA)

Binding properties of sera to SARS-CoV-2 prototype-Beta chimeric RBD-dimer protein were determined by ELISA. 96-well plates (3590; Corning, USA) were coated over-night with 3 μg/ml of prototype-Beta chimeric RBD-dimer protein in 0.05 M carbonate-bicarbonate buffer, pH 9.6, and blocked in 5% skim milk in PBS. Serum samples from macaques were serially diluted and added to each well. The plates were incubated for 2 hours and then washed. The plates were incubated with goat anti-monkey IgG-HRP antibody (Abcam, ab112767), incubated for 1.5 hours and then washed. The plates subsequently developed with 3,3’,5,5’-tetramethylbenzidine (TMB) substrate. Reactions were stopped with 2 M hydrochloric acid, and the absorbance was measured at 450 nm using a microplate reader (PerkinElmer, USA). The endpoint titers were defined as the highest reciprocal dilution of serum to give an absorbance greater than 2.5-fold of the background values. Antibody titer below the limit of detection was determined as half the limit of detection.

#### Challenge of mice with prototype SARS-CoV-2 and Beta variant

To evaluate the protection efficacy of vaccine candidates against prototype SARS-CoV-2, BALB/c mice model transduced intranasally with a recombinant adenovirus recombinant adenovirus 5 expressing human ACE2 (Ad5-hACE2) was used. Immunized BALB/c mice were i.n infected with 8 × 10^9^ vp of Ad5-hACE2. Five days later, the transduced mice were challenged with 5 × 10^5^ TCID_50_ of SARS-CoV-2 (hCoV-19/China/CAS-B001/2020 strain) via the i.n. route. For Beta SARS-CoV-2 challenge experiments, the BALB/c mice were directly infected with 1 × 10^6^ TCID_50_ of Beta SARS-CoV-2 (GDPCC-nCoV84 strain) through the i.n. route. Five days post challenge, all mice were euthanized and necropsied. Lung tissues were collected and split into two parts for virus titration and pathological examination. All mice experiments with SARS-CoV-2 challenge were conducted under ABSL3 facilities.

Mice lung tissues were weighed and homogenized. SARS-CoV-2-specific quantitative reverse transcription-PCR (qRT-PCR) assays were performed using a FastKing One Step Probe RT-qPCR kit (Tiangen Biotech, China) on a CFX96 Touch real-time PCR detection system (Bio-Rad, USA) according to the manufacturer’s protocol. Two sets of primers and probes were used to detect a region of the N gene of viral genome (Chandrashekar et al., 2020) and a region of E gene of subgenomic RNA (sgRNA) of SARS-CoV-2 (Wölfel et al., 2020), respectively, with sequences as follows: gRNA-F, GACCCCAAAATCAGCGAAAT; gRNA-R, TCTGGTTACTGCCAGTTGAATCTG; gRNA-probe, ACCCCGCATTACGTTTGGTGGACC; sgRNA-F, CGATCTCTTGTAGATCTGTTCTC; sgRNA-R, ATATTGCAGCAGTACGCACACA; sgRNA-probe, ACACTAGCCATCCTTACTGCGCTTCG.

To perform the histopathology analysis, mice lung tissues were fixed in 4% paraformaldehyde, dehydrated, embedded in paraffin, and then sectioned. Tissue sections (4 μm) were deparaffinized in xylene and stained with haematoxylin and eosin (H&E) for pathological examination, including peribronchiolitis (inflammatory cells, primarily lymphocytes, surrounding a bronchiole), perivasculitis (inflammatory cells, primarily lymphocytes, surrounding a blood vessel), interstitial pneumonitis (increased thickness of alveolar walls associated with inflammatory cells, primarily neutrophils), and alveolitis (inflammatory cells, primarily neutrophils and macrophages, within alveolar spaces).

#### Challenge of mice with Delta and Omicron SARS-CoV-2 variants

To evaluate the protection efficacy of vaccine candidates against Delta and Omicron variants, the immunized mice were challenged with 6 × 10^5^ TCID_50_ of Delta variant (CCPM-B-V-049-2105-8) or Omicron variant (BA.1, CCPM-B-V-049-2112-18) via the i.n. route. For Delta variant challenge experiments, the BALB/c mice were transduced intranasally with 8 × 10^9^ vp of Ad5-hACE five days before the SARS-CoV-2 infection. Three days post challenge, all mice were euthanized and necropsied, and lung tissues were collected for virus titration and pathological examination. The mice experiments with Delta and Omicron variants challenge were conducted under ABSL3 facilities in IMBCAMS. SARS-CoV-2-specific qRT-PCR assays were performed using TaqMan Fast Virus 1-Step Master Mix kit (Thermo Fisher Scientific, USA) on a CFX384 Touch Real-Time PCR Detection System (Bio-Rad, USA) according to the manufacturer’s protocol. The sequences of primers and probes used in the qRT-PCR assays were same as the above description, except for detecting Omicron variant gRNA with the probe sequence of ACTCCGCATTACGTTTGGTGGACC. Mice lung tissues were stained with H&E for pathological examination.

#### Macaque experiments

Twenty-four rhesus monkeys (n = 12) were immunized with clinical-grade prototype-Beta chimeric vaccine (25 μg antigen + Aluminum hydroxide adjuvant) and placebo (Aluminum hydroxide adjuvant), respectively. Three doses were administrated at days 0, 21 and 42. These monkeys were bled for humoral immunogenicity evaluation before immunization and 14, 35 and 52 days after receiving the first dose.

To evaluate the cellular immune responses elicited by prototype-Beta chimeric vaccine, PBMCs were collected at day 48 and stimulated with peptide pool consisting of 15–18-mers (overlapping by 11 amino acids) and spanning the RBD of prototype SARS-CoV-2. INF-γ, IL-2 and IL-4 ELISpot assays were performed with ELISpot kits according to the manufacturer’s protocols (MabTech, cat#3421M-4APW-10, cat#3445M-4APW-10 and cat#3410-2APW-10).

At the day 56, 57 and 58, animals of prototype-Beta chimeric vaccine group (2 female and 2 male) and sham group (2 female and 2 male) were intranasally and intratracheally challenged with total 1×10^6^ TCID_50_ prototype SARS-CoV-2 (GDPCC-nCoV27), Beta variant (GDPCC-nCoV84) or Delta variant (CCPM-B-V-049-2105-8). Animals were anesthetized for monitoring body weight and temperature before and post SARS-CoV-2 challenge. Body temperature was measured via electronic scale. Anal temperature was measured via electronic thermometer.

Before challenge and at the days 1, 3, 5, 7 post infection, nasal, throat, and anal swabs were collected from anesthetized animals into clean tube and lysed with Trizol. RNA was eluted with RNase/DNase-free distill water and stored at -80°C.

At 7 DPI, animals were euthanized and dissected for pathological examination of lung. Tissue samples were collected from the left lung (upper lobe, middle lobe and inferior lobe) and the right lung (upper lobe, middle lobe, inferior lobe and accessory lobe) for viral load and histopathology. Examination was performed with emphasis on the histopathology of lung. For evaluation of viral load, tissue samples were collected from 6 sites of each lung lope. Total about 100 mg of mixed tissue samples was minced Trizol. RNase/DNase-free distilled water was used to elute RNA, which was stored at -80°C.

One-step qRT-PCR was performed to measure viral genomic RNA (gRNA) with primers and probe paired with viral N gene that is recommended by WHO and China CDC. The sequences of primers and probe were same as those used in the prototype SARS-CoV-2 gRNA titration experiments described above. The reaction conditions and preparation of the standard for N gene were performed according to SOP or the manufacture’s protocols.

#### Histopathology analysis of rhesus macaque lungs

Lung tissue samples of macaques were collected from seven lobes as described above and fixed in neutral formalin. Sections were stained via H&E for histological examination Double-blind evaluation was made on the basis of the histopathological profile of all collected lobe tissues. The histopathological changes of lung, such as inflammation, structure change and hemorrhage, were graded according to the following scoring system.

Score 0 indicates clear structure of alveolar without inflammatory infiltration. Score 1 indicates mild inflammation, slightly widened alveolar septum and sparse mononuclear cells (monocytes and lymphocyte infiltration). Score 2 indicates severe inflammation, thickening of alveolar wall and increased inflammatory infiltration of interstitial monocytes. Score 3-4 indicates alveolar septum widened significantly, increased infiltration of inflammatory cells. Score 5 indicates extensive exudation and widened septum, smaller alveolar cavity, septal bleeding, and infiltration of alveolar cells. Score >5 indicates a large number of cells infiltrated into the alveolar cavity; the alveolar cavity disappeared; the septum fused, and a transparent membrane was formed on the alveolar wall.

At least 5 fields of each section were randomly chosen for evaluation of histopathological changes according to the scoring system above. Histopathological changes of each lobe of lung from every macaque were graded based on thickening or consolidation of pulmonary septum, bleeding of pulmonary septum, infiltration of inflammatory cells, vascular thrombosis and distribution area of dust cells. The total score of each index is the final score of histopathological changes for each lobe of lung. The average of scores from all lobes is referred to the histopathological score for the whole lung of each animal.

### Quantification and statistical analysis

K_D_ values for SPR assays were calculated by the software BIAevaluation Version 3.0 (GE Healthcare) using 1:1 binding model.

Pseudovirus neutralization titer (pVNT_50_) was determined by fitting nonlinear regression curves using GraphPad Prism and calculating the reciprocal of the serum dilution required for 50% neutralization of infection. The values shown are GMT ± 95% CI. The endpoint titer of antigen-binding IgG and 50% neutralization titer of authentic SARS-CoV-2 were shown as GMT ± 95% CI. Details can be found in figures legends.

The NAb titers and pulmonary viral gRNA were analyzed with a linear model using GraphPad Prism. Details can be found in figures legends.

Pooled analyses of pathological scores for lung tissues were shown as means ± SEM. *P*-values were analyzed with two-tailed Mann Whitney test. Details can be found in figures legends.

*P*-values were analyzed with two-tailed Mann Whitney test or two-tailed unpaired t test (ns, p > 0.05; ^∗^p < 0.05; ^∗∗^p < 0.01; ^∗∗∗^p <0.001; ^∗∗∗∗^p < 0.0001). Details can be found in figures legends.

## Data Availability

The cryo-EM maps have been deposited in the Electron Microscopy Data Bank with accession codes EMDB: EMD-33225 (SARS-CoV-2 prototype RBD-dimer bound to CB6 Fab), EMDB: EMD-33234 (SARS-CoV-2 prototype-Beta chimeric RBD-dimer bound to CB6 Fab) and EMDB: EMD-33235 (SARS-CoV-2 Delta-Omicron chimeric RBD-dimer bound to CB6 Fab). All the other data supporting the finding of this study are available within the paper and are available from the corresponding author upon request. This study did not generate unique code.

## Supplemental information

Table S1. Detailed information of rhesus macaques, related to Figure 3
