# Supplementary material for: Protective prototype-Beta and Delta-Omicron chimeric RBD-dimer vaccines against SARS-CoV-2
Source: Cell. 2022 Jun 23;185(13):2265–2278.e14. doi: 10.1016/j.cell.2022.04.029 (PMC9042943; doi:10.1016/j.cell.2022.04.029)
Supplement: Table S1. Detailed information of rhesus macaques, related to Figure 3 [file mmc1.docx]

**Table S1.** **Detail information of rhesus macaques, related to Figure 3**

| **Number** | **Sex** | **Age** | **Immunization** | **Virus challenge** |
| --- | --- | --- | --- | --- |
| 19050 | F | 2.6 | Sham | Prototype SARS-CoV-2 |
| 18086 | F | 2.2 | Sham | Prototype SARS-CoV-2 |
| M13 | M | 2.3 | Sham | Prototype SARS-CoV-2 |
| 19033 | M | 2.6 | Sham | Prototype SARS-CoV-2 |
| 18110 | F | 2.4 | Sham | Beta variant SARS-CoV-2 |
| 18084 | F | 2.2 | Sham | Beta variant SARS-CoV-2 |
| M7 | M | 2.3 | Sham | Beta variant SARS-CoV-2 |
| M9 | M | 2.6 | Sham | Beta variant SARS-CoV-2 |
| 18158 | F | 2.2 | Sham | Delta variant SARS-CoV-2 |
| 18162 | F | 2.2 | Sham | Delta variant SARS-CoV-2 |
| 18113 | M | 2.3 | Sham | Delta variant SARS-CoV-2 |
| M15 | M | 2.3 | Sham | Delta variant SARS-CoV-2 |
| 19174 | F | 2.2 | prototype-Beta chimeric RBD-dimer | Prototype SARS-CoV-2 |
| 19200 | F | 2.4 | prototype-Beta chimeric RBD-dimer | Prototype SARS-CoV-2 |
| M16 | M | 2.4 | prototype-Beta chimeric RBD-dimer | Prototype SARS-CoV-2 |
| 18121 | M | 2.3 | prototype-Beta chimeric RBD-dimer | Prototype SARS-CoV-2 |
| 19192 | F | 2.3 | prototype-Beta chimeric RBD-dimer | Beta variant SARS-CoV-2 |
| 19194 | F | 2.3 | prototype-Beta chimeric RBD-dimer | Beta variant SARS-CoV-2 |
| 19207 | M | 2.2 | prototype-Beta chimeric RBD-dimer | Beta variant SARS-CoV-2 |
| M10 | M | 2.4 | prototype-Beta chimeric RBD-dimer | Beta variant SARS-CoV-2 |
| 18100 | F | 2.4 | prototype-Beta chimeric RBD-dimer | Delta variant SARS-CoV-2 |
| 19176 | F | 2.2 | prototype-Beta chimeric RBD-dimer | Delta variant SARS-CoV-2 |
| 18117 | M | 2.2 | prototype-Beta chimeric RBD-dimer | Delta variant SARS-CoV-2 |
| 18145 | M | 2.3 | prototype-Beta chimeric RBD-dimer | Delta variant SARS-CoV-2 |
